# *SLPI*⁺ AT2‐Like Cells Orchestrate Lung Adenocarcinoma Invasion via Wnt Pathway Activation and Stromal Crosstalk in a Spatially Defined Margin Niche

**DOI:** 10.1002/advs.202516580

**Published:** 2025-11-11

**Authors:** Zhoufeng Wang, Guonian Zhu, Pan Tang, Yan Wang, Wenxin Luo, Wenpeng Song, Zhikang Pan, Bingjie Zheng, YongChao Jiang, Defu Xiao, Xin Jin, Yong Bai, Guowei Che, Weimin Li

**Affiliations:** ^1^ Department of Pulmonary and Critical Care Medicine Frontiers Science Center for Disease‐related Molecular Network West China Hospital Sichuan University Chengdu 610041 China; ^2^ State Key Laboratory of Respiratory Health and Multimorbidity West China Hospital Sichuan University Chengdu 610041 China; ^3^ Precision Medicine Key Laboratory of Sichuan Province West China Hospital Sichuan University Chengdu 610041 China; ^4^ Department of Thoracic Surgery West China Hospital Sichuan University Chengdu 610041 China; ^5^ Lung Cancer Center West China Hospital Sichuan University Chengdu 610041 China; ^6^ BGI Research Shenzhen 518085 China; ^7^ College of Life Sciences University of Chinese Academy of Sciences Beijing 101408 China; ^8^ Department of Biomedical Engineering Southern University of Science and Technology Shenzhen 518055 China; ^9^ BGI Research Chongqing 401122 China; ^10^ State Key Laboratory of Genome and Multi‐omics Technologies BGI Research Shenzhen 518083 China; ^11^ The Innovation Centre of Ministry of Education for Development and Diseases School of Medicine South China University of Technology Guangzhou 510006 China; ^12^ Shenzhen Key Laboratory of Transomics Biotechnologies BGI Research Shenzhen 518083 China

**Keywords:** macrophage, minimally invasive lung adenocarcinoma, SLPI, stemness, tumor microenvironment

## Abstract

The spatial organization of the tumor microenvironment (TME) profoundly influences cancer biology. However, the cell types and spatial distribution driving lung adenocarcinoma (LUAD) invasion remain poorly understood. By integrating spatially resolved transcriptomics with scRNA‐seq data, we identify a novel secretory leukocyte protease inhibitor (SLPI)‐expressing AT2‐like subpopulation that localizes specifically at the invasive tumor margin, which drives the transition from minimally invasive (MIA) to invasive (IA) LUAD. Functional characterization reveals that *SLPI*‐expressing AT2‐like cells upregulated Dickkopf‐1(DKK1), enhancing tumor cell stemness and epithelial‐mesenchymal transition (EMT). Furthermore, spatially co‐localized MRC1‐expressing resident tissue macrophages (RTM‐TAMs) secrete pro‐tumor cytokines upon interaction with *SLPI* ^+^ tumor cells, alongside cancer‐associated myofibroblasts (myo‐CAFs) exhibiting reduced type I collagen production. These TME components establish a pro‐tumorigenic niche and engaged in synergistic interactions with *SLPI* ^+^ AT2‐like cells to facilitate LUAD invasion. These findings reveal the specific cellular composition, spatial architecture, and functional crosstalk between tumor cells and TME subpopulations that orchestrated LUAD progression. The frontier molecular targets at the tumor invasive identified in this study can serve as a basis for developing novel therapeutic targets in the future and assist pathologists in accurately assessing patients' disease progression and survival outcomes.

## Graphical abstract

Integrating spatial transcriptomics and single‐cell RNA sequencing, this study identifies a novel subpopulation of SLPI⁺ AT2‐like cells at the invasive margin of lung adenocarcinoma (LUAD). These cells, through stromal crosstalk and Wnt pathway activation, promote tumor stemness and invasion. The findings highlight *SLPI⁺* AT2‐like cells as potential therapeutic targets for LUAD progression.

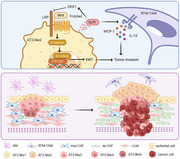

## 1 Introduction

Lung adenocarcinoma (LUAD), the most prevalent subtype of lung cancer,^[^ ^1^, ^2^ ^]^ undergoes a critical progression from minimally invasive adenocarcinoma (MIA), where 5‐year survival following tumor resection approachs 100%, to invasive adenocarcinoma (IA), characterized by significantly worse prognosis and increased recurrence risk.^[^ ^3^, ^4^ ^]^ Despite curative surgical resection, 30–75% of LUAD patients experience disease recurrence.^[^ ^5^ ^]^ The invsasion margin, comprising tumor cell layers at the border of LUAD lesions, has been previously identified as a prognostically significant feature in clinical grading and is implicated in mediating tumor invasion and metastasis.^[^ ^6^, ^7^ ^]^ However, the molecular mechanisms operating at the invasive edge and within other spatially defined tumor  regions remain incompletely understood. Deciphering the specific cell populations that drive invasive progression and elucidating their spatial localization within tumors could provide critical insights into disease mechanisms, revealing potential therapeutic vulnerabilities to halt or decelerate tumor progression.^[^ ^8^ ^]^

Previous efforts employing immunohistochemistry and in situ hybridization to investigate spatial features of LUAD have been constrained by limited throughput and an inability to comprehensively profile the complex tumor microenvironment (TME).^[^ ^9^, ^10^ ^]^ Recent advances in single‐cell RNA sequencing (scRNA‐seq) have enabled the exploration of intratumoral heterogeneity in LUAD.^[^ ^11^, ^12^ ^]^ For instance, studies based on scRNA‐seq have illuminated the roles of stromal and immune cells populations within LUAD tumor and normal tissues.^[^ ^13^, ^14^ ^]^ We previously discovered five distinct malignant cell states in LUAD and traced their progression from normal alveolar type 2 (AT2) cell lineages to tumor cells.^[^ ^15^ ^]^ However, scRNA‐seq lacks the spatial context necessary to correlate transcriptional dynamics with tumor topography. Spatially resolved transcriptomics (SRT) addresses this limitation by capturing gene expression profiles while simultaneously preserving the 2D positional information of cells, thereby enabling holistic characterization of transcriptional heterogeneity within the TME.^[^ ^16^, ^17^ ^]^ In this study, we leveraged high‐resolution SRT to precisely characterize the cellular ecosystem of LUAD and to elucidate the molecular mechanisms underlying tumor invasion.

Here we integrated scRNA‐seq, which identifies the cellular diversity but not the spatial organization within the microenvironment of lung tumors,^[^ ^18^, ^19^ ^]^ with spatially resolved transcriptomics (SRT), which maps cell type localization at micro‐scale resolution.^[^ ^16^, ^20^, ^21^, ^22^, ^23^ ^]^ This complementary approach has proven effective for identifying and spatially localizing cell subtypes that drive invasion and metastasis within the TME.^[^ ^23^, ^24^ ^]^ We aimed to define the molecular signatures distinguishing preinvasive from invasive LUAD and to identify key malignant cell subtypes orchestrating tumor progression. Our study provides insights into invasive cell populations enriched at the tumor margin could serve as actionable therapeutic targets for preventing LUAD progression.

## 2 Results

### 2.1 Single‐Cell and Spatial Transcriptomic Analysis Revealed the Cell Types and Location of LUAD

**Figure 1:**
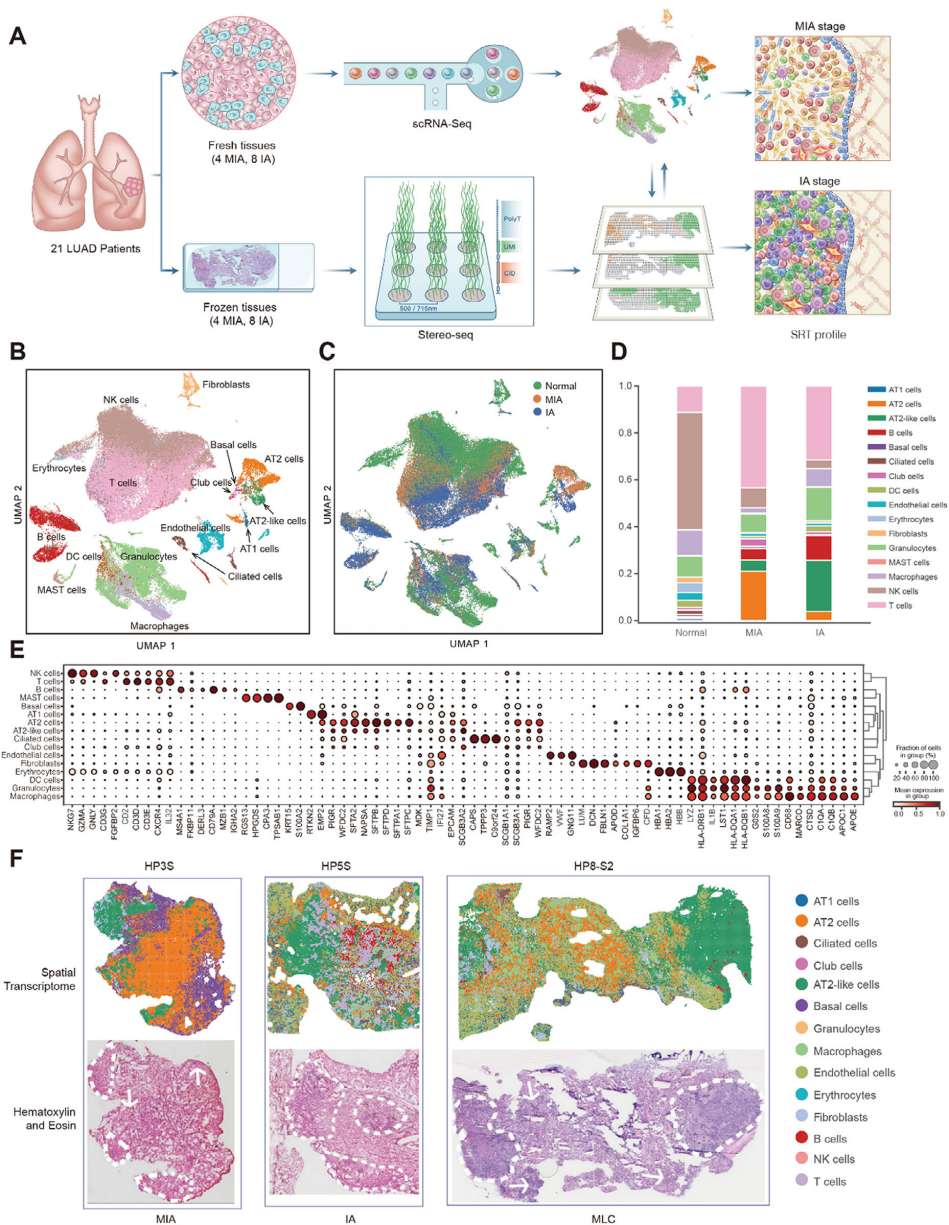
Single‐cell and spatially resolved transcriptomics of cell populations of the tumor microenvironment in minimally invasive and invasive LUAD. A) Schematic overview of the experimental workflow for single‐cell RNA sequencing and spatially resolved transcriptomics (Stereo‐seq) performed on 24 tumors. B) UMAP plot from scRNA‐seq data, colored and annotated to show 16 distinct cell populations. DC, dendritic; NK, natural killer. C) UMAP plot of scRNA‐seq illustrating single‐cell distributions across normal lungs, MIA and IA sample groups. D) Distribution of cell populations in MIA and IA stages of LUAD, compared with normal lung tissues. E) Heatmap depicting the relative expression levels of marker genes across the 16 cell populations. F) Spatial transcriptomics‐based annotation of cell populations. The corresponding HE satined image is shown below, tumor regions are indicated by arrows and dashed lines. MLC: multiple lung cancer.

To elucidate the cellular composition and invasion trajectory in early stage LUAD, we performed scRNA‐seq and Stereo‐seq on 24 tumors from 21 patients (Patient #1‐21), including 8 tumors pathologically diagnosed cases of MIA and 16 cases of IA (Table S1, Supporting Information). Two patients presented with multiple nodules (Figures S1–S3, Supporting Information). These two complementary datasets enabled precise mapping of cell‐type‐specific marker genes expression and spatial localization within the TME (**Figure** **1**A). To investigate how transcriptional changes drive LUAD progression from MIA to IA, we reclustered cells based on their spatial distribution patterns within two tumors situated on slides from patient 8 and 9, as well as a third tumor across three hierarchical levels.

The scRNA‐seq data comprising 61 451 cells were integrated with our previously published LUAD scRNA‐seq dataset to create an unbiased reference expression profile for annotating cell types in spatial transcriptomes.^[^ ^13^ ^]^ This yielded 103 600 high‐quality cells with 27 622 shared genes, from which we identified 16 distinct populations using Scanpy^[^ ^25^ ^]^ based on expression of canonical marker genes (Figure 1B–E, Table S2, Supporting Information). Uniform manifold approximation and projection (UMAP) analysis revealed clusters including ciliated cells (*CFAP53* and *SNTN*), club cells (*SCGB1A1* and *SCGB3A1*), AT1 cells (*RTKN2* and *EMP2*), AT2 cells (*HHIP*, *SFTPC* and *SFTPA*), AT2‐like cells (*NAPSA*, assigned as malignant cell types) and basal cells (*KRT5* and *S100A2*), fibroblasts (*LUM* and *DCN*), endothelial cells (*VWF*) and immune cells such as T cells (*CD3E* and *IL‐32*), NK cells (*NKG7* and *GZMA*), B cells (*CD79A*), macrophages (*MARCO* and *APOE*), granulocytes (*LILR4*) and dendritic cells (*S100A8* and *S100A9*). We found that cell types proportions varied across the spectrum from normal lung tissues to MIA and finally IA stage (Figure 1D).

To characterize the spatial transcriptional landscape of LUAD, we employed Stereo‐seq to generate SRT datasets, achieving a median of 696–5809 transcripts (corresponding to 531–2952 genes) per spot across tissue slides (Figure S4A–C, Supporting Information). Leveraging the scRNA‐seq data as a reference, we applied cell2location to deconvolve multi‐cell spots into cell‐type abundances and assigned each spot to the cell type with the highest abundance, revealing 14 cell types across these slides. These cell types exhibited notable variations in proportions between MIA and IA tissues, with AT2‐like cells demonstrating pronounced enrichment in IA tumors (Figure S4D, Supporting Information). Malignant cell positions and spatial distributions were validated through hematoxylin and eosin (HE) staining, with tumor boundaries outlined by pathologists based on HE images (Figure 1F; Figures S3 and S4E–H, Supporting Information). Additionally, we performed conjoint analysis on tissue sections from two patients with multifocal nodules and designated cancerous regions, yielding activity maps that aligned with annotated or histologically identifiable structures (Figure S4G,H, Supporting Information).

### 2.2 Distribution of Major Cell Types Between the MIA and IA Stage of Tumors

**Figure 2:**
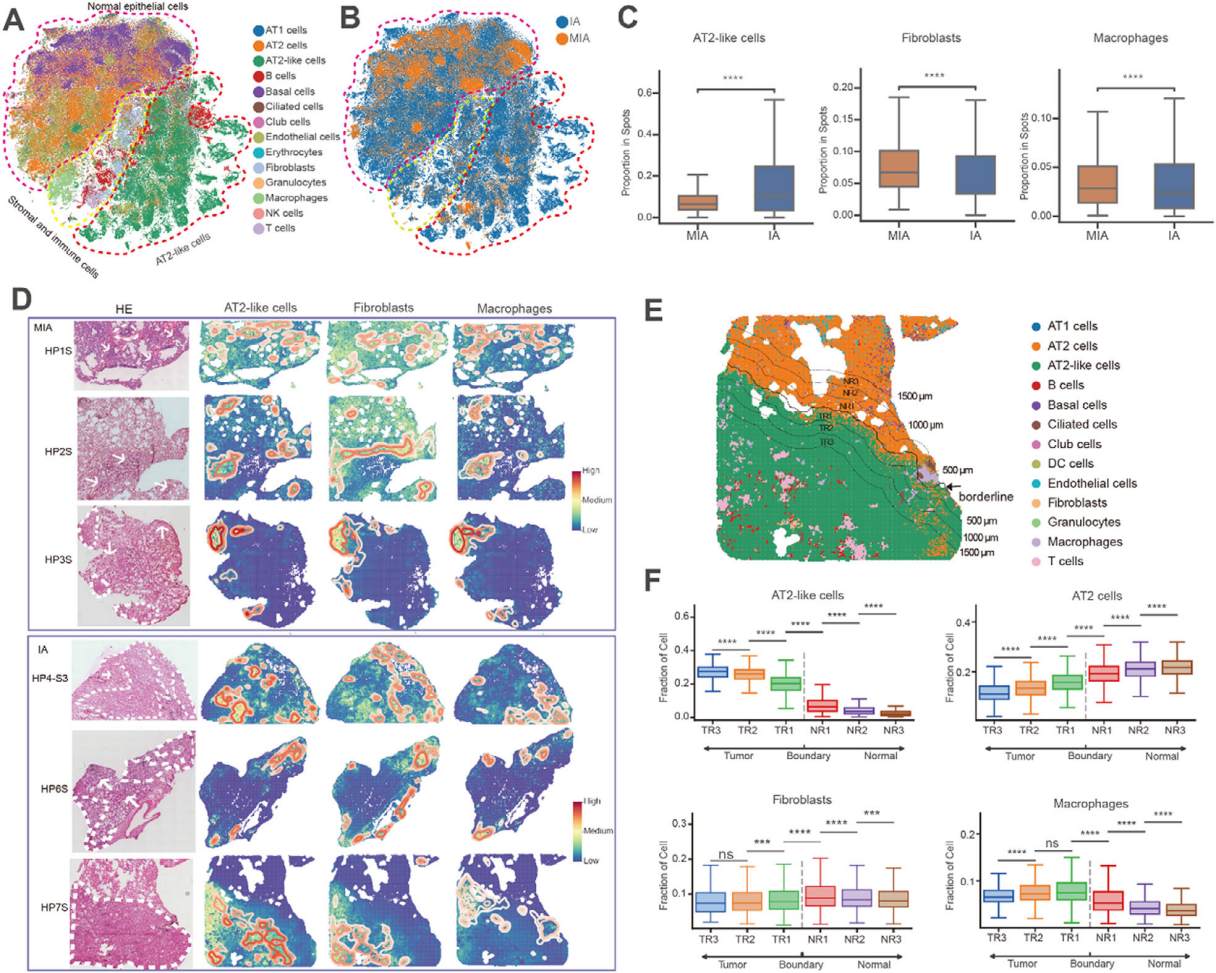
Comparison of the relative abundance and spatial distribution of major cell populations in MIA and IA LUAD tumors. A) t‐SNE visualization of all spots from SRT data. Cell types for each spot were inferred through cell2location deconvolution, using scRNA‐seq data as reference. NK, natural killer. B) t‐SNE visualization of all spots, colored by MIA and IA stages, based on SRT data. C) Proportions of AT2‐like cells, fibroblasts and macrophages in MIA (*n* = 4 replicates) and IA (*n* = 11 replicates) samples from SRT data. D) Spatial density maps illustrate the numbers of spots assigned to AT2‐like cells, fibroblasts or macrophages via deconvolution in three MIA and three IA tumors. E) Borderline demarcating tumor tissue from neighboring normal tissue (from a representative LUAD patient, patient #7). The adjacent region was divided into six 500‐µm‐wide zones by defining parallel offsets extending 500, 1000, and 1500 µm perpendicularly on either side. F) Relative abundances of cells across 500‐µm‐wide zones at the tumor interface in a sample from LUAD patient 7. Abundances align with those depicted in panel (E). Boxplot elements: center line, median; box limits, upper and lower quartiles; whiskers, 1.5 × interquartile range (IQR). Statistical significance assessed by two‐sided Wilcoxon rank‐sum test (C, F). **** *p* < 0.0001. ns, not significant.

To investigate TME dynamics across 15 spatial transcriptomics datasets spanning MIA to IA stages, we compiled all annotated spots *n* = 431 558 spots, median 27 109 per slide (range: 8387—49 675) from Stereo‐seq slides. The t‐distributed Stochastic Neighbor Embedding (t‐SNE) analysis delineated three primary cell clusters among the 14 cell types, including normal epithelial cells, AT2‐like cells, and stromal/immune cells (**Figure** **2**A,B). Pronounced elevation of specific cell‐type marker genes in designated clusters validated the accuracy of cell type annotations for the SRT spots (Figure S5A–C, Supporting Information). AT2‐like cells exhibited significantly higher relative abundance in IA tissues compared to MIA, whereas fibroblasts and macrophages were preferentially enriched in MIA tissues (*p* < 0.0001; Figure 2C,D). Collectively, these observations suggest that a sustained escalation in AT2‐like cells may facilitate the invasive process of LUAD.

To examine how changes in cell populations impact tumor progresses from MIA to IA phenotype, we conducted Gene Ontology (GO) enrichment analysis on genes differentially expressed in AT2‐like cells, fibroblasts and macrophages. In AT2‐like cells (e.g., *S100A8*, *S100A9*, *CIT*), MIA‐stage genes showed enrichment for ion channel activity, while IA‐stage genes were linked to phagocytosis and immune response processes (Figure S5D,G, Supporting Information). Fibroblasts (e.g., *IGHG4, FOS, EMP2*) exhibited IA‐stage enrichment in genes related to extracellular matrix organization (Figure S5E,H, Supporting Information). Macrophages displayed MIA‐stage enrichment in receptor‐ligand activation signaling (Figure S5F,I, Supporting Information). Overall, these patterns align with the known immune and metabolic heterogeneity of lung cancer.^[^ ^26^ ^]^

A key insight from this study underscores the importance of examining differentially expressed genes in AT2‐like cells between MIA and IA tumors. Kyoto Encyclopedia of Genes and Genomes (KEGG) pathways analysis demonstrated enrichment of IL‐17 signaling and epithelial‐mesenchymal transition (EMT) pathways among IA‐stage genes (Figure S5J,K, Supporting Information). Gene set variation analysis (GSVA) revealed MIA‐stage enrichment in G2M checkpoint and Wnt/β‐catenin signaling components, contrasted by predominant EMT signaling at the IA stage (Figure S5L, Supporting Information). Together, these findings link invasive LUAD progression to dysregulated gene expression in cell cycle regulation, IL‐17 signaling, and EMT pathways.

### 2.3 Spatial Transcriptional Features of Cell Types at the Margin of LUAD Tumors

To assess the spatial organization of cell populations and gene expression at the margins of LUAD tumors, we defined a borderline to separate tumor from adjacent normal tissue and partitioned the surrounding region into six 500‐µm‐wide bands (see Methods). We found that AT2 cells prevailed in normal tissues, whereas AT2‐like cells were significantly enriched within tumor tissues (*p* < 0.0001; Figure 2E,F). Expression of the AT2 canonical marker surfactant protein‐C (*SFTPC*) was minimal in tumor and tumor edge areas, where alveolar structures were absent (Figure S5M, Supporting Information). By contrast, SFTPC levels were elevated in normal tissue distal from the tumor, primarily localizing to peripheral alveoli, and markedly higher than that in the tumor edge area. These observations accord with epithelium cancerization, in which epithelial cells involves cellular dedifferentiation.^[^ ^13^, ^27^, ^28^ ^]^

We also observed notable enrichment of fibroblasts and macrophages adjacent to invasive tumors, with fibroblasts significantly accumulating within regions on the tumor side and macrophages on the normal side (*p* < 0.0001; Figure 2E,F). These observations potentially indicated specialized roles for these cell populations in LUAD progression. In addition, dendritic cells and T cells were enriched on the tumor side, while B cells and NK cells predominated on the normal side (Figure S5N, Supporting Information). Altogether, these findings indicate that the TME around the tumor margin areas comprises a specific border microenvironment association with LUAD progression.

### 2.4 Subtypes of AT2‐Like Cells May Contribute to Invasion of LUAD

**Figure 3:**
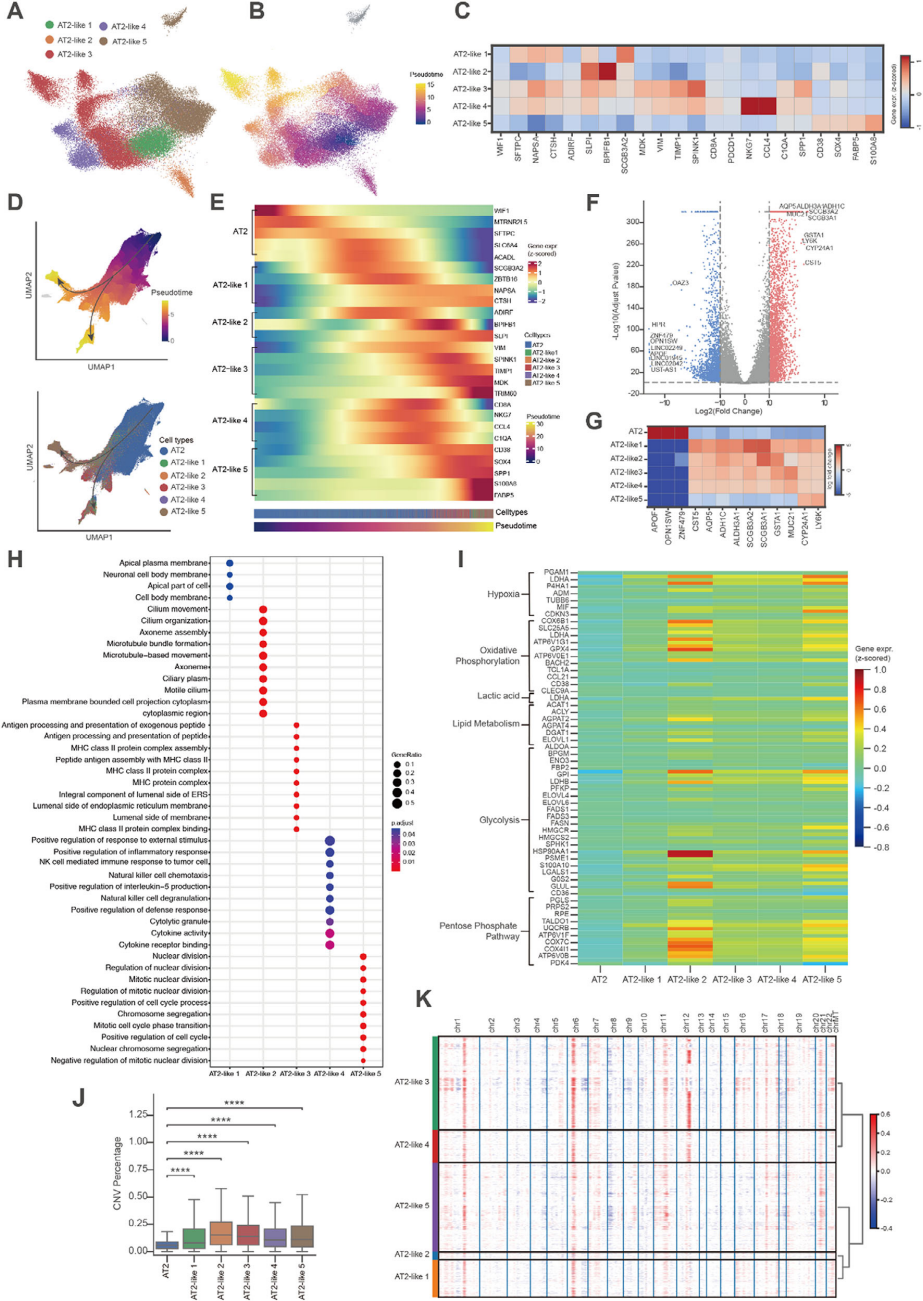
Trajectory of AT2 and AT2‐like cells in LUAD tumors based on spatial transcriptomics. A) UMAP visualization depicting spots assigned to five AT2‐like subtypes according to marker gene expression profiles. B) UMAP visualization illustrates five subclusters of AT2‐like cells color‐coded by pseudotime. C) Heatmap displaying expression patterns of genes exhibiting the greatest variability across the five AT2‐like subtypes. D) Inferred trajectory of AT2 and AT2‐like cells, differentiated by pseudotime (upper panel) and AT2‐like subtypes (lower panel), generated using Molecle3 algorithm. Analysis included normal AT2 spots (*n* = 115 020; range, 1815—24 859; median, 5429) and AT2‐like subtype spots (*n* = 51 193; range, 93–8195; median, 2681). E) Heatmap of differentially expressed genes (log_2_ fold change>2, adjusted *p* < 0.05). colored according to z‐scored gene expression. F) Volcano plot highlighting genes with differential expression between AT2 cells and AT2‐like 1 subtypes. G) Heatmap of differentially expressed genes between AT2 and AT2‐like 1 cells from panel (F). H) Gene Ontology term enrichment of genes differentially expressed across AT2‐like subtypes 1–5. I) Heatmap depicting expressions of metabolic‐related genes in AT2 cells and AT2‐like subtypes. J) Boxplot showing the prevalence of copy number variations in AT2 and AT2‐like cells. Boxplot elements: center line, median; box limits, upper and lower quartiles; whiskers, 1.5 × IQR. Statistical significance assessed by two‐sided Mann‐Whitney test. **** *p* < 0.0001. K) Heatmaps display patterns of copy number variations across AT2‐like subtypes.

We next explored the heterogeneity of cancer cells within the transcriptomic spots from SRT data. Leveraging specifically upregulated genes from our previous work,^[^ ^13^ ^]^ we precisely categorized AT2‐like cells into five distinct subtypes (**Figure** **3**A,C). To investigate their spatial evolution during LUAD progression, we performed pseudotime analysis using Molecle3.^[^ ^29^, ^30^ ^]^ This unveiled a developmental trajectory in which AT2 cells progress to AT2‐like 1 cells, which subsequently branch into AT2‐like 2–5 subtypes (Figure 3B,D). Inter‐samples analysis revealed that all five AT2‐like subtypes coexisted across tumor stage, with AT2‐like 1 prevalent in MIA, AT2‐like 3 in IA, and AT2‐like 5 enriched in both (Figure S6A,B, Supporting Information).

To elucidate the functional roles of the five AT2‐like cell subtypes, we examined each major tumor cell population in our SRT data. We found that the AT2‐like 4 subtype was linked to genes typically expressed in lymphocytes (e.g., *CCL4*, *CD8A*, *NKG7*), whereas the AT2‐like 5 subtype was tied to myeloid‐associated genes (e.g., *C1QA*, *S100A8*, *SPP1*) (Figure S6C, Supporting Information). Using our newly generated scRNA‐seq data, we reclustered and annotated these cancer populations, confirming  that AT2‐like 4 and 5 cells expressed immunity‐related genes (Figure S6D,E, Supporting Information). These observations were consistent with previous studies showing that lung AT2 cells can express immunity‐related genes, with enhanced proliferative capacity yet limited antigen presentation ability.^[^ ^31^, ^32^ ^]^

We then identified differentially expressed genes (DEGs) with dynamic patterns across pseudotime (*q*‐value < 0.05) and categorized them into six clusters (corresponding to AT2 cells and AT2‐like 1–5 subtypes). These genes were arranged along the pseudotime axis, with top 5 genes per cluster ranked and visualized in a diffusion map (Figure 3E). This analysis revealed elevated expression of tumor suppressor genes such as *ZBTB16* and *ACADL* in AT2‐like 1 cells,^[^ ^33^, ^34^ ^]^ which diminished rapidly as the pseudotime trajectory advanced. Additionally, we observed a progressive loss of expression of the classical AT2 cell marker *SFTPC* during LUAD progression.^[^ ^13^ ^]^ Comparative analysis of differentially expressed genes between AT2 and AT2‐like 1 cells revealed upregulated metabolic genes (*AQP5*, *ALDH3A1* and *ADH1C*) in the AT2‐like 1 subtype, with their expression levels declining along the pseudotime (Figure 3F,G). Enrichment analysis indicated that AT2‐like 2 genes were enriched in KEGG metabolic pathways and GO terms for microtubule bundle formation and movement that implied rapid proliferation of AT2‐like 2 cells, whereas AT2‐like 3 genes were enriched in KEGG immune responses signaling pathways and GO for MHC protein complexes terms that suggested the activation of immune‐related signaling pathways (Figure 3H). Collectively, these results suggested that the heightened proliferation and metabolic activity in AT2‐like 2 cells may drive LUAD advancement.

We next examined cancer‐related metabolic shifts across the five AT2‐like subtypes relative to AT2 cells, the AT2‐like 2 subtype manifested the most pronounced upregulation of various metabolic pathways, with several key genes involved in the pentose phosphate pathway (PPP)^[^ ^35^ ^]^ (Figure 3I, Figure S6F,G, Supporting Information). Consistently, spots with elevated expression score for TKT were spatially enriched at the leading edges of tumor margin (Figure S6H,I, Supporting Information). These findings validated that AT2‐like 2 cells, characterized by enhanced PPP activity, may constitute a unique tumor cell population in LUAD. To evaluate copy number variation (CNV) profiles among the five different AT2‐like subtypes derived from SRT data, we performed chromosomal analysis on AT2 cells and AT2‐like cells using *inferCNV*. This approach enabled high‐resolution delineation of clonal evolution across subtypes throughout the tissue. Notably, AT2‐like 2 cells exhibited a significantly greater CNV burden than AT2 cells and the other AT2‐like subtypes (*p* < 0.0001; Figure 3J), featuring gains on chromosome 12 and losses on chromosome 8 (Figure 3K). These observations indicated that AT2‐like 2 cells may represent a highly malignant population marked by high genomic instability.^[^ ^17^, ^36^ ^]^

### 2.5 SLPI Contributes to Tumor Invasion During LUAD Progression

**Figure 4:**
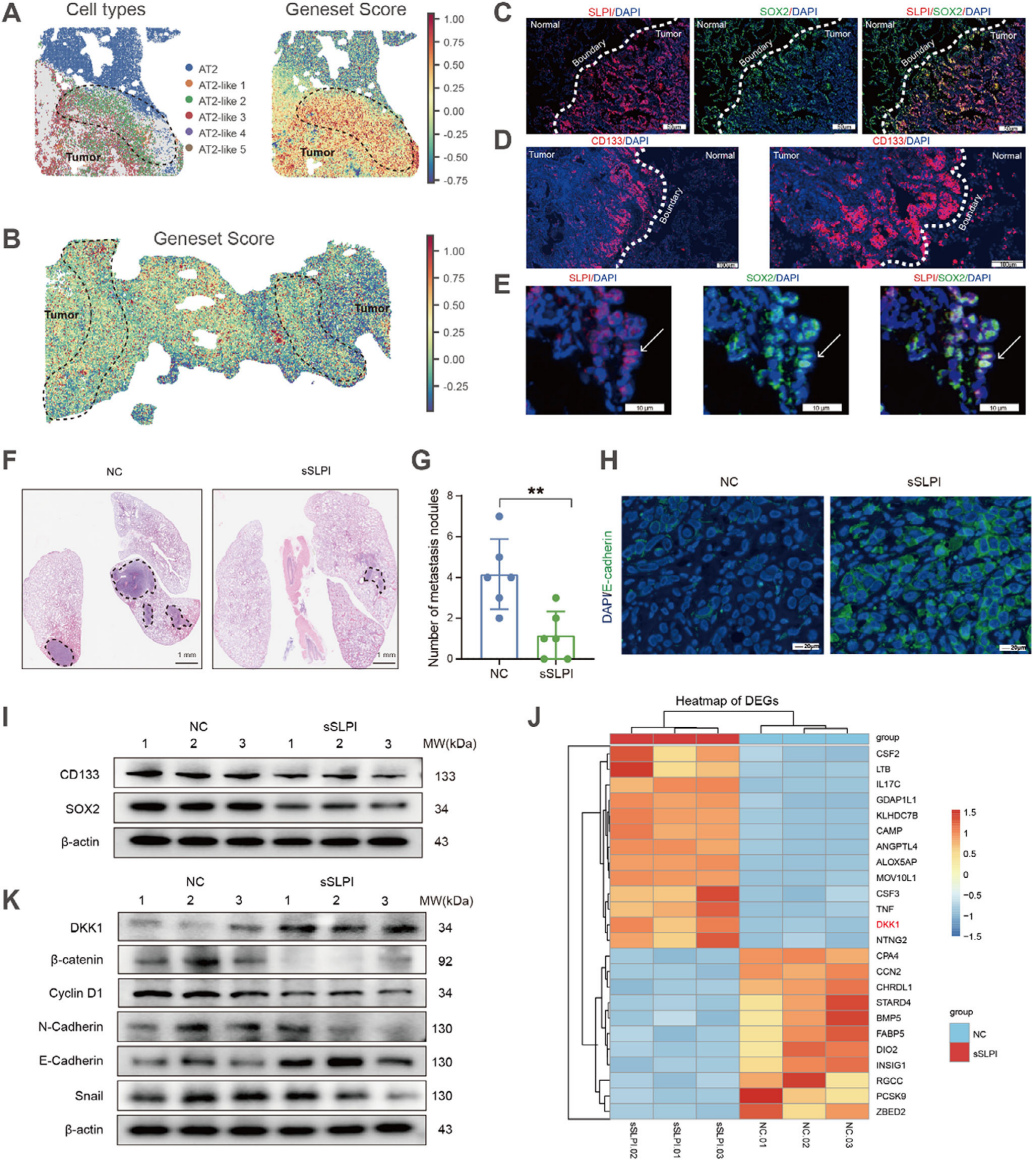
SLPI contributes to invasiveness at the tumor margin. A) Spatial distribution of spots assigned to AT2 and AT2‐like subtypes (left panel) alongside gene‐set scores (right panel) in the IA stage from patient 7. Dotted line highlights an area enriched for AT2‐like 2 subtype at the tumor margin. B) Spatial distribution of spots based on gene‐set score in an IA sample from patient 8, featuring two tumors within a single section. Dotted lines illustrate areas enriched for AT2‐like 2 subtype near the tumor periphery. C) IF staining for SLPI and SOX2 in cells within the invasive region of IA‐stage LUAD samples. Dashed lines represent the border (*n* = 3). D) IF staining demonstrates elevated CD133 expression in tumor cells within the invasive region of IA‐stage LUAD samples (*n* = 3) (left: Low magnification, right: High magnification). Dash lines represent the border. E) IF staining revealing co‐expression of SLPI and SOX2 in tumor cells within the invasive region of IA‐stage LUAD samples. Dashed lines represent the border (*n* = 3). F) HE staining images of mouse lungs 8 weeks after tail‐vein injection of 2 × 10^6^ PC9 cells (NC) and SLPI‐shRNA PC9 cells (sSLPI). Black outlines denote tumor formation regions (*n* = 6). G) The number of lung‐metastasis nodules on each lung surface. Data represents as mean ± standard deviation (S.D). Statistical significance assessed by unpaired t test (*n* = 6). ** *p* < 0.01. H) IF staining for E‐cadherin in xenografts excised from mice against (*n* = 3). Scale bar = 20 µm. I) WB analysis of CD133 and SOX2 expression in PC9 cells (NC) and SLPI‐shRNA PC9 cells (sSLPI) (*n* = 3). J) Differential gene expression patterns identified via RNA‐seq in PC9 Cells (NC) and SLPI‐shRNA PC9 Cells (sSLPI) (*n* = 3). K) WB analysis of Wnt/ pathway‐related indicators in PC9 cells (NC) and SLPI‐shRNA PC9 cells (sSLPI) (*n* = 3).

We observed elevated expression of secretory leukocyte protease inhibitor (*SLPI)* and BPI fold‐containing family B member 1 (*BPIFB1*) in AT2‐like 2 cells within SRT data (Figure 3C). Notably, *SLPI* displayed prominent and selective expression in AT2‐like cells, especially at the tumor invasive front. Previous studies have demonstrated that SLPI promotes tumor cell migration, invasion, and immune evasion by modulating protease activity and extracellular matrix remodeling in breast cancer.^[^ ^37^ ^]^ Based on these findings, we hypothesized that SLPI may play a critical and conserved role in promoting tumor invasiveness in LUAD. To test this hypothesis, we computed a gene set score for AT2‐like 2 cells and mapped their spatial distribution within tumor tissues. AT2‐like 2 cells exhibiting elevated scores were primarily positioned at the leading edges of tumor margins (**Figure** **4**A,B, Figure S7A, Supporting Information), and showed upregulation of stemness‐associated genes, such as *CD133*, *SOX2*, *SCGB3A2*, and *SFTPB* (Figure S7B, Supporting Information). Notably, AT2‐like 2 cells were present in 13 out of 15 tumors analyzed, with each of these tumors harboring at least 3 such spots (Figure S7C, Supporting Information). These patterns suggest that AT2‐like 2 cells may represent a stemness‐ and EMT‐ related subpopulation that persists across different tumor stages. For validation, we performed multiplexed immunofluorescence (IF) staining for SLPI, CD133, and SOX2 in LUAD tissues, revealing colocalized overexpression of these proteins specifically at the tumor invasive front (Figure 4C–E). Collectively, these results indicate that the spatial enrichment of *SLPI*‐expressing AT2‐like 2 cells at tumor margins strongly correlates with invasive LUAD progression, potentially mediated through their stem‐like properties and vascular mimicry capacity.

Next, we explored the potential direct involvement of SLPI in promoting invasion in LUAD. We first investigated the role of SLPI as a potential driver of LUAD invasion in terms of cellular behaviors. We found that knocking down SLPI in the LUAD cell line PC9 inhibited its proliferation, migration and invasion in vitro (Figure S7D–F, Supporting Information). Similarly, the knockdown of SLPI (sSLPI) suppressed PC9 xenograft growth in nude mice (Figure S7G–K, Supporting Information). In metastasis models involving intravenously injected PC9 tumor cells, fewer pulmonary metastatic foci were observed in sSLPI group (Figure 4F,G). Further IF staining also confirmed E‐cadherin downregulation in the sSLPI mouse model, consistent with our results in vitro (Figure 4H). Based on our preceding findings, we further validated at the cellular level that CD133 and SOX2 expression was significantly reduced in sSLPI cells (Figure 4I), supporting our hypothesis that SLPI may mediate its pro‐tumorigenic effects on LUAD by modulating cellular stemness.

To elucidate the mechanism underlying SLPI‐mediated invasion, we performed RNA sequencing (RNA‐seq) analysis to examine differences in stemness‐related gene expression between PC9 cells and SLPI‐knockdown PC9 cells. Our analysis revealed prominent upregulation of DKK1 (Dickkopf‐1,LogFC = 2.92) in the sSLPI group, which was further confirmed by Western blot (Figure 4J). Given the well‐established role of DKK1 as a Wnt/β‐catenin pathway antagonist,    and its documented importance in tumor progression, invasion, and metastasis.^65^ We therefore conducted a thorough assessment of Wnt/β‐catenin pathway activity and found that SLPI depletion substantially decreased β‐catenin and its downstream target *cyclin D1*, while concurrently increasing the epithelial marker *E‐cadherin*. Conversely, mesenchymal markers, including *N‐cadherin* and *Snail*, were significantly downregulated in sSLPI cells (Figure 4K). Collectively, these findings suggest that overexpression of SLPI promotes cancer cells to gain stemness through activation of DKK1‐dependent Wnt/β‐catenin pathway and facilitating tumor invasion via EMT potentiation.

### 2.6 Tissue‐Resident Macrophages Provide a Pro‐Tumoursigenic Niche in LUAD Progression

**Figure 5:**
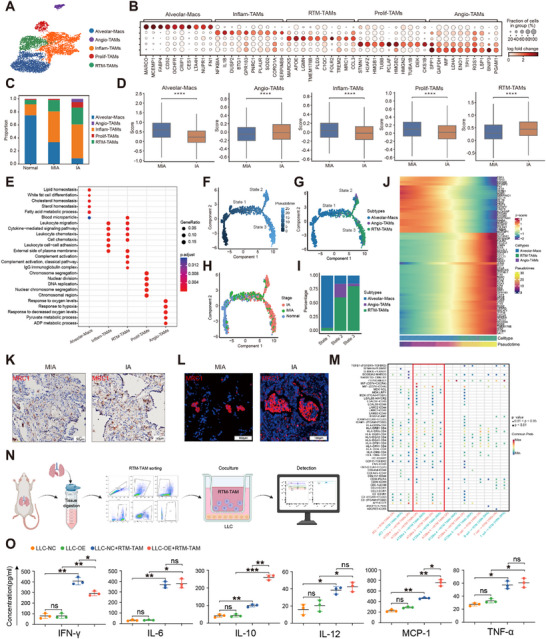
Tissue‐resident macrophages establish a pro‐tumorigenic niche in LUAD tumors. A) UMAP plot showing five distinct clusters among 6142 macrophages from scRNA‐seq data, with colors representing cell types. B) Dotplot illustrating genes with differential expression patterns across five macrophage subtypes. C) Proportions of macrophage subtypes in MIA, IA and normal lung tissues from scRNA‐seq data. D) Boxplots displaying GSVA scores for each macrophage subtype in MIA and IA tumors based on scRNA‐seq data. Boxplot elements: center line, median; box limits, upper and lower quartiles; whiskers, 1.5 × IQR. Statistical significance assessed by two‐sided Mann‐Whitney U test. **** *p* < 0.0001. E) GO enrichment analysis of differentially expressed genes among five macrophage subtypes from SRT data. F, G, H) Inferred developmental trajectories of macrophage subtypes along pseudotime, distinguished by pseudotime progression(F), subtypes(G), and tissue types spanning MIA, IA and normal lung tissues from scRNA‐seq data (H). I) Proportion of three macrophage subtypes across different trajectory states in the scRNA‐seq data. J) Heatmap depicting differentially expressed genes among macrophage subtypes in the scRNA‐seq dataset, sorted according to pseudotime. K) IHC staining for MRC1 expression in tumor tissues from MIA and IA samples. Scale bar: 50 µm. L) IF staining for MRC1 (red) in tumor tissues from MIA and IA stages in LUAD patients. Nuclei were stained with DAPI (blue). Scale bar: 500 µm. M) Dotplot illustrating ligand‐receptor interactions between diverse cell types and receptors on RTM‐TAMs. Dot size reflects statistical significance (large dots: *p* < 0.01; small dots 0.01< *p* < 0.05), with only large dots shown. Analyses were performed using CellChat (v1.6.1) and CellChatDB.human (v1) database. N) Schematic overview of the protocol for isolating and sorting RTM‐TAM cells from mouse lung tissues. O) CBA assessment of cytokine levels in cell culture supernatants. LLC‐NC and LLC‐OE (SLPI‐overexpressing) cells were cultured alone, and LLC‐NC/LLC‐OE were co‐cultured with RTM‐TAM cells. Cytokine levels including IFN‐γ, IL‐6, IL‐10, IL‐12, MCP‐1, and TNF‐α were analyzed using CBA and flow cytometry. Statistical significance assessed by two‐way ANOVA test (*n* = 3). * *p* < 0.05, ** *p* < 0.01, ***p < 0.001; ns, not significant. IFN, interferon; IL, interleukin; MCP, monocyte chemoattractant protein; TNF, tumor necrosis factor.

To investigate macrophages changes across LUAD progression, we analyzed the scRNA‐seq data from 6142 macrophages and identified five distinct subtypes based on their marker genes (**Figure** **5**A,B, Figure S8A,B, Supporting Information). These substypes encompassed alveolar macrophages (Alveolar‐Macs), inflammatory cytokine‐enriched macrophages (Inflam‐TAMs), pro‐angiogenic macrophages (Angio‐TAMs), resident‐tissue macrophages (RTM‐TAMs), and proliferating macrophages (Prolif‐TAMs).^[^ ^38^ ^]^ Notably, Prolif‐TAMs were identified through the high expression of marker *Ki67* and cell cycle‐related pathway gene *PCLAF*.^[^ ^39^ ^]^ Angio‐TAMs expressed *SPP1*, *ENO1*, and *PGAM1*, which were angiogenesis‐related genes as described previously.^[^ ^40^ ^]^ We also detected RTM‐TAMs highly expressed canonical macrophage genes such as *MRC1*, *C1QA*, *CD68* and *APOE* but lacked typical monocyte signature.^[^ ^41^ ^]^ Proportions of these five macrophage subtypes varied distinctly between MIA, IA and normal lung tissues (Figure 5C,D). Specifically, Alveolar‐Macs were enriched in normal lung tissues, whereas Angio‐TAMs, Inflam‐TAMs and RTM‐TAMs exhibited a gradual rise in prevalence as tumor advanced from MIA to IA. Genes strongly expressed in RTM‐TAMs were enriched in GO terms related to leukocyte migration, while those expressed in Alveolar‐Macs were in lipid homeostasis, and those in Angio‐TAMs were in pathways of response to hypoxia (Figure 5E). These findings may suggest the dichotomous functional phenotypes of macrophages in the LUAD TME.

We then performed pseudotime trajectory analysis using Monocle2 on Alveolar Macs, RTM‐TAMs and Angio‐TAMs, which uncovered structured gene expression dynamics along the pseudotime trajectory (Figure 5F–H). This analysis delineated a developmental path primarily originating from Alveolar Macs and bifurcated into either the RTM‐TAMs or the Angio‐TAMs terminal differentiation cluster. Our examination highlighted shifts in the proportions of these subtypes across the trajectory from MIA to IA (Figure 5I). Specifically, Alveolar‐Macs predominated in early‐stage MIA tumors (state 1), whereas RTM‐TAMs and Angio‐TAMs became more prevalent in advanced stages (states 2–3). In state 2, the rise in Angio‐TAMs correlated with tumor progression. Meanwhile, state 3 featured a dominance of RTM‐TAMs with elevated expression of *CCL4*, *CCL13*, and *TMEM176B* (Figure 5J), lending support to the concept that M2‐like macrophages foster a type 2 inflammatory milieu in the LUAD tumor microenvironment during progression.

Previous studies have indicated that RTM‐TAMs originated from Alveolar‐Macs and promote tumor progression.^[^ ^38^, ^41^ ^]^ In the TCGA data set, the *MRC1* signature score with higher expression also showed a significant correlation with worse outcomes.^[^ ^42^ ^]^ To further explore the biological functions of RTM‐TAMs in the invasion of LUAD, we calculated GSVA scores of differentially expressed genes for the five macrophage subtypes derived from SRT data (Figure S8C, Supporting Information). We found that RTM‐TAMs were localized close to the tumor cells and predominantly distributed at the front of tumor margins (Figure S8D, Supporting Information). The IHC and IF staining results revealed that RTM‐TAMs were enriched close to the tumor, which resembled human granuloma lesions (Figure 5K, L). These results suggested that RTM‐TAMs might also be associated with AT2‐like 2 cells at the front of the tumor, playing a role in shaping the TME during the progression of LUAD.

To examine communications between RTM‐TAMs and other cell components in LUAD, we inferred putative cell‐cell interactions using CellChat. As expected, the analysis suggested that RTM‐Macs strongly interacted with AT2 cells and AT2‐like 2 cells in both MIA and IA stages (Figure S8E,F, Supporting Information). This evidence could substantiate the notion that AT2‐like 2 cells interact with RTM‐TAMs to promote tumor growth. To further characterize potential specific interaction between AT2‐like2 cells and RTM‐TAMs in distinct border regions, we identified two active signaling pathways involving macrophages in tumors of MIA stage, mediated by *KIT* (CD117) and *ANGPTL*, whereas 17 active signaling pathways mediated by *SPP1*, *THBS* and *MPZ* of macrophages in IA stage (Figure S8G, Supporting Information). *KIT* (CD117), which is known to be highly expressed in lung cancer,^[^ ^43^ ^]^ is a receptor tyrosine kinase that regulates proliferation, differentiation and adhesion of cells. Analysis of ligand‐receptor pairs showed that pathways mediated by *MIF*, *MHC‐II*, *SPP1* and *FN1* escalated during the progression from MIA to IA stage, likewise for those involving *CDH1* and *VCAM* (Figure S8H,I, Supporting Information), indicating their potential role in regulating anti‐tumor responses.

To further validate the spatial and functional association between AT2‐like 2 cells and RTM‐TAMs, we integrated observations from Figure 2E where macrophages were enriched in the tumor margin adjacent to the tumor mass, overlapping with the spatial distribution of AT2‐like 2 cells. CellChat analysis further suggested potential ligand‐receptor interactions between these two cell populations. Subsequently, we examined the communication between different cell types and RTM‐TAMs, and found that signaling appeared to be particularly strong between the AT2‐like 2 subtype and the *MIF*‐*CD74* and *LGALS9*‐*CD44* subtypes of RTM‐TAMs. These macrophages communicated specifically with AT2‐like 2 subtypes via FN1‐CD44 and FN1‐(ITGA4+ITGB1) (Figure 5M). These results suggest that ligand‐receptor interactions involving RTM‐TAMs are important in the progression of LUAD.

To experimentally address these ligand‐receptor interactions, we isolated RTM‐TAMs from mouse lung tissues via enzymatic digestion and flow cytometric sorting (Figure S8J, Supporting Information). The sorted RTM‐TAMs were then co‐cultured with or without SLPI‐overexpressing LLC cells for 48 h (Figure 5N), followed by cytokine profiling of the conditioned media (Figure S8K, Supporting Information). Notably, co‐culture with SLPI‐overexpressing cells resulted in significant upregulation of MCP‐1 and IL‐10, accompanied by a marked reduction in IFN‐γ levels (Figure 5O). These findings indicated that SLPI‐mediated signaling suppressed T cell function and inhibited macrophage polarization toward an anti‐tumor M1 phenotype, thereby fostering a pro‐tumorigenic microenvironment that promoted LUAD progression. This experimental validation complemented our spatial transcriptomic and computational analyses, establishing a mechanistic link between SLPI‐expressing AT2‐like 2 cells and RTM‐TAMs in the formation of an immunosuppressive niche at the tumor invasive front.

### 2.7 Distribution of Three Distinct Fibroblast Subsets with Multi‐Faceted Roles in LUAD Ecosystem

**Figure 6:**
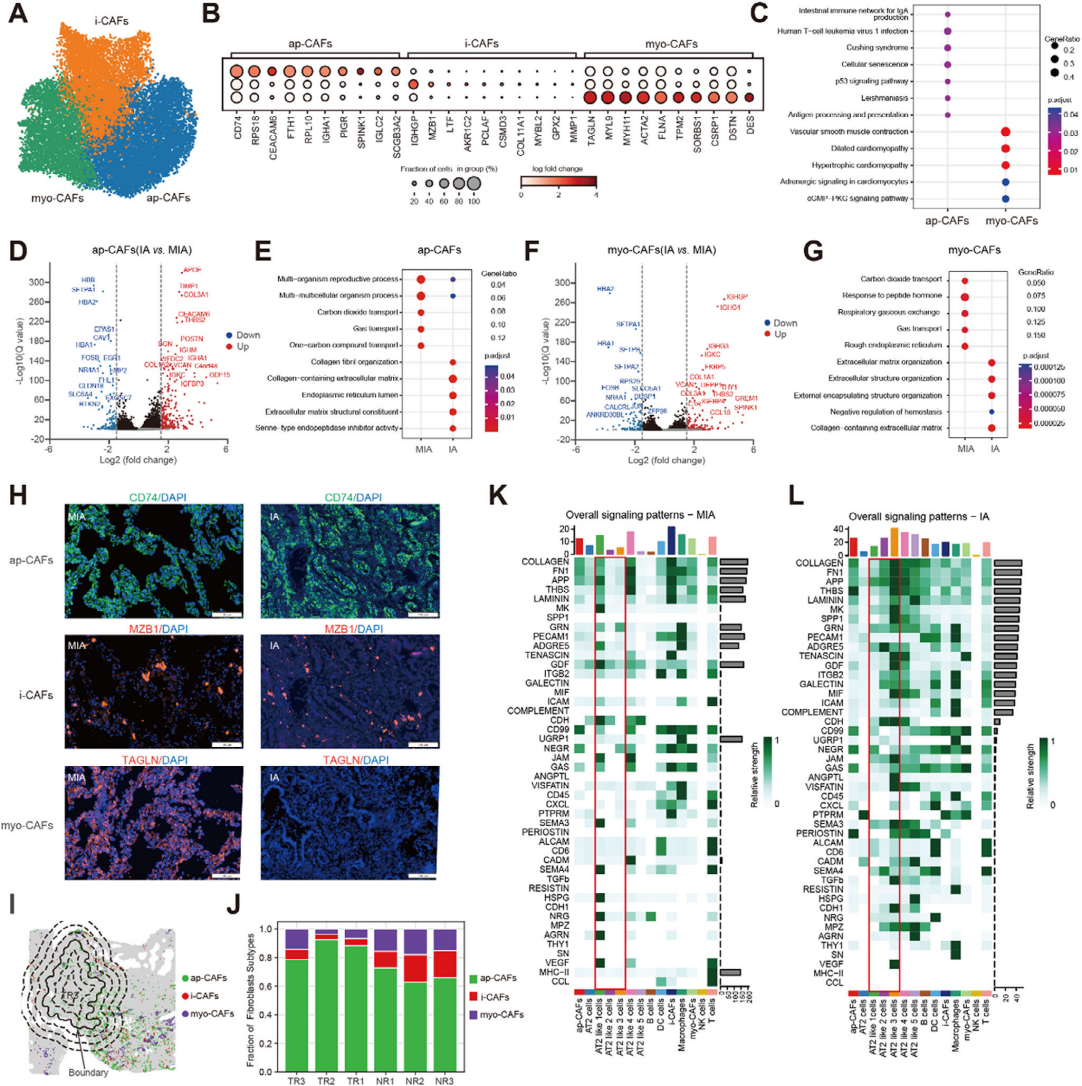
Spatial distribution and transcriptomic profiles of fibroblast subtypes in LUAD tumors. A) UMAP analysis delineating three fibroblast subtypes derived from spatial transcriptomics data. B) Dot plot visualizing genes with differential expression patterns across the three subtypes. C) KEGG pathway enrichment analysis of genes differentially expressed between antigen‐presenting fibroblasts (ap‐CAFs) and myofibroblasts (myo‐CAFs). D, F) Volcano plots highlighting differential gene expression of ap‐CAFs (D) and myo‐CAFs (F) between MIA and IA stages. Each dot represents a gene: red dots denote upregulation in IA (*q*‐value<0.05), blue dots denote upregulation in MIA (*q‐*value<0.05), and black dots represent genes not meeting the fold‐change threshold (i.e., |log_2_ fold changes|<1.5). E, G) GO enrichment analysis of differentially expressed genes in ap‐CAFs (E) and myo‐CAFs (G) between MIA and IA stages. H) IF staining for ap‐CAFs (CD74, green), i‐CAFs (MZB1, red) and myo‐CAFs (fibronectin, red) in tumor tissues from MIA and IA stages in LUAD patients. Nuclei were stained with DAPI (blue). Scale bars: 100 µm. I) Proportions of fibroblasts across sequential 500‐µm bands (extending 3000 µm axially) at the tumor interface from LUAD patient 2. Bands are defined as follows: NR1(0–500 µm), NR2(500–1000 µm) and NR3 (1000–1500 µm) toward the normal tissue side; TR1 (0–500 µm), TR2 (500–1000 µm) and TR3 (1000–1500 µm) toward tumor side. J) Proportion of the three fibroblasts subtypes across the six bands as shown in panel (I). K, L) Heatmaps depicting probabilities of selected signal pathways mediated by ligands from diverse cell populations to receptors in MIA (K) and IA (L) tumors.

Considering the plasticity in fibroblast abundance and phenotype in the microenvironment of various types of tumors,^[^ ^44^ ^]^ we next examined the transcriptomic profiles of fibroblasts in LUAD. Through this analysis, we identified three fibroblast subtypes, including antigen‐presenting fibroblasts (ap‐CAFs), inflammatory fibroblasts (i‐CAFs), and myofibroblasts (myo‐CAFs) (**Figure** **6**A,B, Figure S9A,B, Supporting Information). Although i‐CAFs were enriched on the tumor side in conjunction with myo‐CAFs, clustering analysis using established i‐CAF markers (Figure 6B) failed to reveal distinct separation or functionally unique subpopulations compared to other fibroblast subtypes. While we recognize that i‐CAFs may still play a role in the TME, the absence of well‐defined functional clusters in our dataset constrained our ability to explore their specific effects. Our analysis showed that ap‐CAFs typically expressed the invariant chain CD74, which was essential for MHC class II transport from the rough endoplasmic reticulum to the late endosome.^[^ ^45^ ^]^ Spatial profiling revealed that ap‐CAFs, marked by CD74, were enriched in the tumor interior, while myo‐CAFs (α‐SMA⁺) localized preferentially at the tumor margin near normal tissue (Figure S9C, Supporting Information). Genes related to the KEGG pathways of immune response and antigen presentation were highly expressed in ap‐CAFs, in contrast to those associated with vascular smooth muscle signaling in myo‐CAFs (Figure 6C).

To comprehensively characterize fibroblast subtype at the population level, we performed differential gene expression analysis between MIA and IA stages (Figure 6D–G). This revealed elevated expression of EMT‐associated genes, such as *APOE*, *TIMP1*, and *COL3A1*, in ap‐CAFs during progression to the IA stage. These observations aligned with prior findings indicating that ap‐CAFs stem from alveolar type 2 (AT2) cells and display enhanced invasive and metastatic potential as tumors evolve.^[^ ^46^, ^47^ ^]^ Meanwhile, myo‐CAFs upregulated *IGHGP*, *IGHG4* and *COL1A1* in IA‐stage tumors, which were involved in the organization of the extracellular matrix (Figure S9D, Supporting Information). The analysis of scRNA‐seq data revealed that myo‐CAF derived collagen I altered the chemokine profile of cancer cells to block the recruitment of myeloid‐derived suppressor cells and allow antitumor B cell immunity.^[^ ^48^ ^]^

Spatial transcriptomics showed that ap‐CAFs and i‐CAFs exhibited greater prevalence in the IA‐stage tumors, whereas myo‐CAFs were more abundant in the MIA‐stage tumors (Figure S9E, Supporting Information). Myo‐CAFs have been reported to be transient during tumor progression, and myo‐CAFs subtypes lost their dominance as other major CAF subsets‐expressing genes linked to growth factor signaling and inflammation‐increased in the late stage of tumors.^[^ ^49^ ^]^ We verified the accumulation of myo‐CAFs in the vicinity of tumors through IF staining and assessed the surrounding spatial transcriptional characteristics (Figure 6H). These findings were consistent with our previous scRNA‐seq observations, which highlighted an enrichment of myo‐CAFs during the initial stages of LUAD, especially in the atypical adenomatous hyperplasia (AAH), adenocarcinoma in situ (AIS), and MIA stages.^[^ ^13^ ^]^ We additionally examined the spatial organization of the three fibroblast subtypes in tumors‐proximal areas via contour‐guided zonal segmentation (Figure 6I). Quantitative analysis revealed that myo‐CAFs and i‐CAFs were preferentially enriched in regions adjacent to the tumor boundary, while ap‐CAFs predominated in more distal, normal tissue–adjacent zones (NR1–NR3) (Figure 6J). This implied that myo‐CAFs may exhibit selective positioning near malignant cells and develop specialized contractile properties.^[^ ^50^ ^]^ Thereby, myo‐CAFs potentially facilitate extracellular matrix remodeling amid LUAD advancement to enhance tumor cell motility and dissemination.

Our preceding observations indicated that both AT2‐like 2 cells and myo‐CAFs were enriched in the front of tumors.^[^ ^12^ ^]^ Therefore, we explored whether AT2‐like 2 cells engage in crosstalk with these fibroblasts and if myo‐CAF depletion might facilitate AT2‐like 2 cell invasiveness. CellChat analysis indeed demonstrated heightened interactions between myo‐CAFs and other cellular components during the transition from MIA to IA stages (Figure S9F,G, Supporting Information). By summing up the probabilities of information flow in different pairwise communications, we identified 28 active signaling pathways involving fibroblasts in IA‐stage tumors, mediated by CACM, CLEC, CDH5, and APP, in contrast to the absence of such networks in MIA‐stage tumors (Figure 6K,L, Figure S9H,I, Supporting Information). Evaluation of potential ligand‐receptor pairs revealed that myo‐CAFs appeared to communicate with AT2‐like 2 subtypes via *COL1A1‐SDC4* and *COL1A1‐CD44* in MIA‐stage tumors. *SDC4* is highly relevant to tumor development and metastasis, with its upregulation documented in diverse malignant tumors, such as renal cell carcinoma, melanoma and breast carcinoma^[^ ^51^, ^52^, ^53^ ^]^ Our data illustrated a reduction in myo‐CAF tumor cell crosstalk from MIA to IA stages, characterized by decreased engagement via *SDC4* and *CD44* on malignant cells. This decline in COL1A1‐ligand interactions could result from lowered type I collagen levels, thereby potentially accelerating tumor advancement. Conversely, we observed that i‐CAFs communicated with AT2‐like 1 subtypes in MIA‐stage tumors, whereas ap‐CAFs interacted with AT2‐like 3 cells in IA‐stage tumors (Figure S9J, Supporting Information). Collectively, these findings implied that fibroblast‐tumor cell communications in LUAD may correlate with shifts in gene expression across these subpopulations.

## 3 Discussion

**Figure 7:**
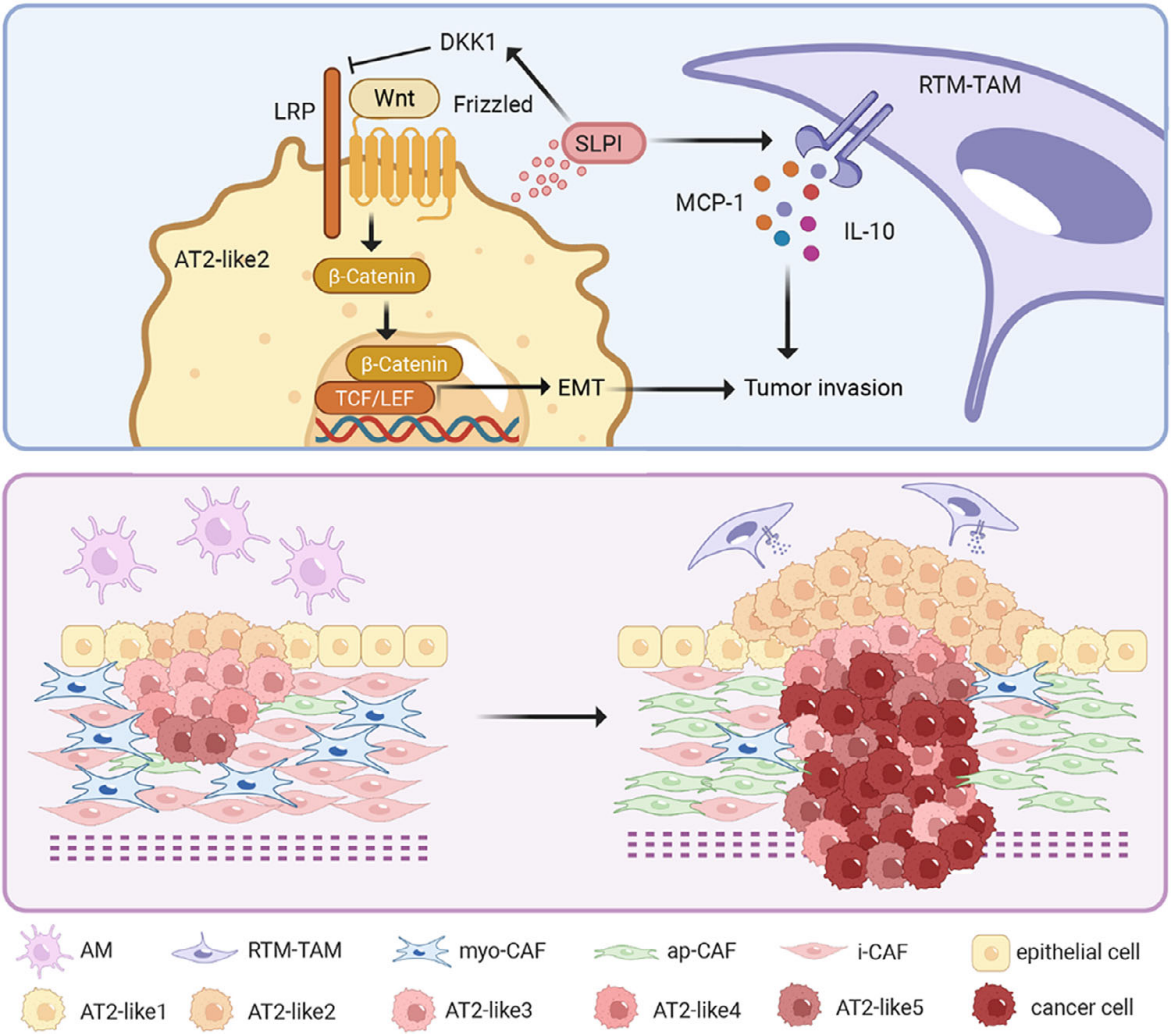
Illustrative overview of cellular compositions and spatial reorganization during LUAD invasion. SLPI‐expressiing AT2‐like2 cells could facilitate activation of the Wnt pathway, leading to elevated stemness and EMT, enhancing  tumor cell invasiveness (top). Specific cellular subpopulations and their spatial localization that are proposed to contributed to invasion of LUAD (bottom).

The integration of single‐cell RNA sequencing and spatial transcriptome profiling provides unprecedented molecular resolution into the mechanisms of LUAD progression. Through comprehensive bioinformatic analysis complemented by in vitro cell culture and in vivo mouse xenograft experiments, we identify *SLPI*‐expressing AT2‐like 2 subtypes enriched at the tumor leading margin as key drivers of invasive progression. These cells promote stemness acquisition through DKK1‐dependent activation of the Wnt/β‐catenin pathway while facilitating tumor invasion via EMT potentiation. Concurrently, the *MRC1^+^* RTM‐TAM cells establish a type 2 inflammatory microenvironment and secrete the immunosuppressive cytokine IL‐10, which synergizes with AT2‐like 2 cells to create a pro‐tumorigenic niche. Additionally, the decline in the production of type 1 collagen by myo‐CAFs contributed to an immunosuppressive microenviroment. Collectively, these spatially coordinated cellular interactions converge to promote LUAD progression (**Figure** **7**). The identification of *SLPI* ^+^ AT2‐like 2 cells as pivotal mediators of tumor invasion warrants further mechanistic investigation and represents a promising avenue for therapeutic intervention in the future.

The present study confirms and substantially extends our previous work linking AT2‐like cells to the development and progression of LUAD.^[^ ^13^ ^]^ In contrast to prior single‐cell studies that focused predominantly on advanced‐stage tumors with high heterogeneity, we examined early‐stage LUAD, enabling robust identification of five distinct epithelial subtypes with greater transcriptional clarity. Critically, we discovered that *SLPI* ^+^ AT2‐like 2 subtypes were specifically enriched at the tumor invasive margin, where their stem‐like properties position them as key drivers of tumor invasion. Functional validation demonstrated that SLPI knockdown significantly suppressed LUAD tumor growth in vivo, establishing a causal role beyond mere association. Moreover, SLPI expression may serve as a clinically actionable prognostic marker for stratifying LUAD patients at elevated risk of invasive progression. While our findings highlight *SLPI* ^+^ AT2‐like 2 cells as critical mediators of tumor advancement, the mechanistic relationship between pentose phosphate metabolism and stemness maintenance in these cells warrants further investigation. Collectively, our results identify SLPI as a potential therapeutic target for intercepting LUAD invasion and progression.

Our experiments, together with our previous studies, illuminate distinct and context‐dependent roles for tumor‐associated macrophage subsets in LUAD pathogenesis. We observed that RTM‐TAMs progressively accumulated during the transition from MIA to IA and localized preferentially to the tumor invasive front. Mechanistically, our co‐culture experiments revealed that SLPI‐overexpressing cells reprogram RTM‐TAMs to secrete MCP‐1 and IL‐10 cytokines, establishing a type 2 inflammatory microenvironment while suppressing anti‐tumor IFN‐γ production. This SLPI‐mediated cytokine polarization likely reinforces the pro‐tumorigenic niche, facilitating EMT and driving tumor invasion.^[^ ^54^ ^]^ In contrast, myo‐CAFs exhibited opposing dynamics, with their abundance declining during MIA‐to‐IA progression. Our data suggest that myo‐CAFs exert tumor‐restraining functions during early tumorigenesis through secretion of type I collagen, which maintains extracellular matrix integrity and inhibits invasion. The observed depletion of myo‐CAFs during the invasive transition likely diminishes this structural barrier via reduced collagen deposition, thereby permitting tumor expansion. This ECM‐mediated regulatory mechanism contrasts with the immune‐suppressive functions attributed to myo‐CAFs in other tumor contexts,^[^ ^55^, ^56^ ^]^ highlighting tissue‐specific and stage‐dependent roles for stromal cell populations in cancer progression.

Several limitations warrant consideration when interpreting our findings. We analyzed surgical specimens from different LUAD patients using scRNA‐seq and SRT, introducing potential inter‐patient variability. Ideally, a comprehensive analysis of LUAD progression would include comparisons across the full histological spectrum from AAH and AIS through MIA to IA. However, AAH and AIS lesions are typically too small to yield sufficient tissue for spatially resolved transcriptomic profiling following diagnostic sampling, precluding their inclusion in this study.

Despite these constraints, our integrated spatial and single‐cell analyses reveal at least three coordinated mechanisms that may drive LUAD invasion. First, we define an “invasive zone” at the tumor leading margin enriched for stem‐like, *SLPI* ^+^ AT2‐like 2 cells, which not only initiate invasion but also reprogram neighboring RTM‐TAMs to secrete pro‐tumorigenic cytokines (MCP‐1, IL‐10) while suppressing anti‐tumor IFN‐γ production, thereby establishing an immunosuppressive niche. Second, high MRC1 expression by RTM‐TAMs sustains a type 2 inflammatory microenvironment that reinforces cytokine‐mediated immunosuppression and facilitates invasion. Third, progressive depletion of myo‐CAFs during MIA‐to‐IA transition diminishes type I collagen deposition (e.g., COL1A1), weakening ECM constraints and enabling tumor cell migration. The spatial heterogeneity inherent to LUAD,^[^ ^57^ ^]^ with intermixed tumor, stromal, and normal tissue compartments, has long complicated mechanistic studies of disease progression. Our spatially resolved approach begins to disentangle these complexities, providing a foundation for targeted interventions to intercept invasive progression. Moving forward, comprehensive characterization of LUAD pathogenesis will require integration of complementary omics modalities, including genomics, epigenomics, proteomics, and metabolomics, to fully capture TME plasticity across disease stages. Such multi‐dimensional approaches, combined with longitudinal sampling strategies and advanced spatial profiling technologies with subcellular resolution, will be essential for translating these mechanistic insights into clinical applications that halt or reverse LUAD progression.

In summary, through integrated spatiotemporal transcriptomic profiling, single‐cell sequencing, and histopathological correlation, we have generated a comprehensive cellular atlas of LUAD that delineates gene expression patterns, spatial organization, and intercellular communication networks across tumor core, invasive margin, and adjacent normal regions. This high‐resolution mapping enabled refined molecular subtyping of malignant epithelial cells and identification of *SLPI* ^+^ AT2‐like 2 cells as critical drivers of invasion at the tumor leading edge. The molecular signatures and spatial biomarkers defined here provide a framework for developing targeted therapeutic strategies to intercept LUAD progression and offer pathologists objective molecular criteria for more accurate prognostic stratification and clinical decision‐making.

## 4 Experimental Section

### Clinical Samples

The clinical samples in this study were prospectively collected from patients diagnosed with lung adenocarcinoma (LUAD) at West China Hospital of Sichuan University (Chengdu, China) from 2019 to 2022, none of whom had received any medication for tumor treatment. Biopsies of tumors and adjacent normal lung tissue were obtained during surgery. All diagnoses were verified through examination of biopsies by a board‐certified pathologist, and the disease was staged according to the TNM system.^[^ ^58^ ^]^ The acquisition and analysis of patient‐derived samples obtained approval from the Institutional Review Board affiliated with West China Hospital of Sichuan University (approval 2 020 232), and patients consented to their samples and anonymized medical data to be analyzed and published for research purposes.

### Sample Collection

Tumors and adjacent normal lung tissues were obtained during surgery. For spatial transcriptomics analyses, all individual tumor samples were snap‐frozen in Optimum Cutting Temperature (OCT) compound (catalog no. 4583, Sakura) and maintained at −80 °C for long‐term preservation until utilization. The specimens were collected and embedded in OCT within half an hour ex vitro. Freshly excised tissues that were destined for single‐cell RNA sequencing were soaked in Dulbecco's Modified Eagle Medium (Gibco, Gaithersburg, MD, USA) supplemented with 10% fetal bovine serum (FBS) and processed for sequencing within 1 h after collection.

### Immunofluorescence Staining of Clinical Samples

Tissues were embedded in paraffin, sectioned to a thickness of 5 µm, subjected to antigen retrieval through heating for 30 min in a microwave in EDTA (pH 9.0), normal anti‐goat serum was used for blocking (45 min), and subsequent overnight incubation at 4 °C was performed with primary antibodies directed against the following proteins: CD74 (1:500; catalog no. HA601117, HuaBio), MZB1 (1:200; catalog no. 11454‐1‐AP, Proteintech), α‐SMA (1:200; catalog no. ab7817, Abcam), TAGLN (1:500; catalog no. ab155272, Abcam), COL1A2 (1:1000; catalog no. 14695‐1‐AP, Proteintech), AGER (1:500; catalog no. ab216329, Abcam), SFTPC (1:100; catalog no. ab90716, Abcam), CD86 (1:400; catalog no. bs‐1035R, Bioss), MRC1 (1:400; catalog no. ab64693, Abcam), SLPI (1:500; catalog no. bs‐6849R, Bioss), CD133 (1:500; catalog no. 66666‐1‐Ig, Proteintech) and SOX2 (1:500; catalog no. 66411‐1‐Ig, Proteintech). Next, slices were incubated with appropriate secondary antibodies in the dark for 1 h. Nuclei were stained with DAPI, and sections were imaged using a VS200 slide scanning system (Olympus).

### Immunohistochemistry of Clinical Samples

Tissues were fixed immediately after collection in 4% paraformaldehyde overnight, sectioned to a thickness of 4 µm, heat‐mediated antigen retrieval was performed on the samples in citrate buffer (pH 6.0), prior to blocking in normal goat serum and overnight incubation at 4 °C with primary antibodies targeting Ki67 (1:500; catalog no. 12 202, Cell Signaling Technology) or SLPI (1:500; catalog no. bs‐6849R, Bioss). The sections were washed, incubated with appropriate secondary antibodies, and exposed to diaminobenzidine (Zhongshan Golden Bridge, Beijing, China) for color development. Sections were counterstained with hematoxylin, dehydrated, cleared, and examined under an upright optical microscope (Nikon).

### Single Cell Transcriptomic Sequencing

Single‐cell transcriptomic sequencing was performed as follows. Fresh tissue specimens were dissected into fragments smaller than 0.5 mm^3^ using sterile scalpels. These fragments were then incubated in 8 mL of pre‐warmed Hank's Balanced Salt Solution (HBSS) containing collagenase I (1 mg mL^−1^) and collagenase IV (0.5 mg mL^−1^) in 15‐mL conical tubes (BD Falcon). Digestion was carried out for 30 min at 37 °C under constant rotation using a Tube Revolver (Thermo Fisher Scientific, USA). The resulting suspension was filtered through a 70‐µm nylon mesh and centrifuged at 500×g. The pellet was treated with red blood cell (RBC) lysis buffer (Sigma) for 5 min at room temperature, followed by another centrifugation step. The final cell pellet was resuspended in phosphate‐buffered saline (PBS) containing 0.04% FBS.

Single‐cell suspensions were loaded onto a Chromium Single Cell Chip (10× Genomics) according to the manufacturer's instructions. ≈5000 cells per sample were targeted for encapsulation along with barcoded Gel Beads. Following cell capture, mRNA was reverse‐transcribed into barcoded cDNA, which was subsequently amplified and used to construct sequencing libraries. All samples from the same patient were processed together on the Chromium Controller (10× Genomics), and libraries were prepared in a single batch to minimize technical variation. Sequencing was performed on an Illumina NovaSeq 6000 platform with the following read structure: an 8‐base index read, a 26‐base read 1 (containing cell barcode and UMI), and a 98‐base read 2 (transcript sequence). Quality control of sequencing data included exclusion of cells expressing fewer than 200 or more than 2500 genes, cells with over 5% of transcripts derived from mitochondrial genes, and genes detected in fewer than three cells.

The scRNA‐seq data were preprocessed using the standard pipeline in Scanpy^[^ ^25^ ^]^(v1.9.1). Stringent quality control were applied, excluding low quality cells with fewer than 200 or more than 2500 expressed genes, greater than 5% expressed mitochondrial gene, or genes expressed in fewer than three cells per sample. Retained gene counts per cell were normalized to the library size and log‐transformed. We selected 1500 highly variable genes, computed z‐scores, and scaled the gene expression data to achieve a mean of zero and variance of one, with values clipped standard deviations exceeding 10. Principal component analysis (PCA) was performed, and the top 40 principal components per cell were used for Leiden clustering at a resolution of 0.15. Differentially expressed genes across the resulting clusters were identified using the “scanpy.tl.rank_genes_groups()” function in Scanpy. Clusters were then annotated as distinct cell populations based on the identified differentially expressed genes and known marker genes.^[^ ^13^ ^]^

### Spatial Transcriptomic Sequencing

DNA nanoballs (DNBs) were prepared following the Stereo‐seq protocol^[^ ^23^ ^]^ by synthesizing random 25‐bases coordinate identifier (CID) oligonucleotides, circularizing them with T4 DNA ligase, and performing rolling circle amplification in conjunction with splint oligonucleotides. These DNBs were subsequently deposited onto patterned chips sized 65 × 65 mm^2^. The CID sequences for each spatial position were established via single‐end sequencing employing the SE25 approach on a DNBSEQ‐Tx21 sequencer (MGI Research). Poly (T) oligonucleotides and 10‐based molecular identifier (MID) oligonucleotides were annealed and joined to the DNBs on the chip, producing capture probes that incorporated a 25‐bp CID barcode, a 10‐bp MID tag, and a 22‐bp poly(T) tail. The CID sequences and their associated coordinates for individual DNBs were ascertained using the manufacturer's designated base‐calling procedure for the DNBSEQ‐T7 sequencer. Post‐sequencing, the capture chip was divided into 10 × 10 mm^2^ sub‐chips, and any redundant CID sequences from non‐contiguous positions were eliminated. For robust annotation and evaluation with sufficient genes, transcripts from identical genes were combined into non‐overlapping 50‐µm‐diameter bins (bin100, 100 × 100 DNB positions), which we designated as spots.

Tissues embedded in OCT compound were cut crosswise into 10‐µm‐thick sections with a CM1950 cryostat (Leica, Wetzlar, Germany), positioned on compact capture chips and glass sides, incubated at 37 °C for 8 min, sterilized in methanol, and held at −20 °C for 30 min. Sections on glass slides underwent hematoxylin‐eosin (HE) staining, and both slide‐ and chip‐mounted sections were imaged using a VS200 microscope (Olympus, Japan).

Tissue samples on capture chips were treated for permeabilization with 0.1% pepsin (Sigma‐Aldrich, St. Louis, MO, USA) in 0.01 M HCl (pH 2.0) at 37 °C for 18 min, followed by rinsing in 0.1× SSC buffer (Thermo Fisher Scientific, USA) containing 0.05 U µL^−1^ RNase inhibitor (New England Biolabs, Ipswich, MA, USA). RNA from the treated tissues was bound by the DNBs on the chips and converted to cDNA via reverse‐transcribed at 42 °C for 1 h, utilizing a mixture of 10 U µL^−1^ reverse transcriptase, 2 U µL^−1^ RNase inhibitor, 1 mM dNTPs, 1 M betaine PCR additive, 7.5 mM MgCl_2_, 5 mM DTT, 2.5 µM Stereo‐TSO, and 1× First‐Strand buffer (BGI). The tissue was then rinsed twice with 0.1× SSC buffer, enzymatically cleared using tissue removal buffer (STOmics, 1 000 028 505) at 55 °C for 30 min, and rinsed twice more with 0.1× SSC buffer. cDNA was detached from the chips through overnight incubation at 55 °C in 3 mL cDNA release solution (STOmics, 1 000 028 512), succeeded by a single rinse with 3 mL nuclease‐free water. The cDNA was refined using 0.8 × Ampure XP Beads (Vazyme Biotech, Nanjing, China) and amplified with KAPA HiFi Hotstart ReadyMix (Roche, Basel, Switzerland), 0.8 µM primers, and the thermal profile: 95 °C for 5 min; 15 cycles of 98 °C for 20 s, 58 °C for 20 s, and 72 °C for 3 min, followed by 72 °C for 5 min. Amplified products were refined with 0.6 × Ampure XP Beads, and their concentration was measured using a Qubit dsDNA Assay Kit (Thermo Fisher Scientific, USA).

A 20‐ng portion of cDNA was enzymatically cleaved with Tn5 transposase at 55 °C for 10 min, with the reaction halted by introducing 0.02% SDS and gently agitation at 37 °C for 5 min. The 25 µL cleaved fragments were amplified in a 100‐µL reaction mix including 0.3 µM Stereo‐Library‐F primer, 0.3 µM Stereo‐Library‐R primer, 1× KAPA HiFi Hotstart ReadyMix, and nuclease‐free water, under the thermal cycling conditions: one cycle at 95 °C for 5 min; 13 cycles at 98 °C for 20 s, 58 °C for 20 s, and 72 °C for 30 s, followed by 72 °C for 5 min. PCR products were purified using Ampure XP Beads for DNBs (0.6× and 0.2×) and subjected to 100‐bp paired‐end sequencing on a DNBSEQ‐Tx21 sequencer.

### Cell Type Deconvolution for Spatial Transcriptomics

We utilized cell2location^[^ ^59^ ^]^ (v0.8a0) to infer cell‐type compositions within multi‐cell spots, thereby projecting cell types identified from scRNA‐seq onto the spatial transcriptomics at multi‐cellular resolution. Prior to analysis, spots with gene counts below the fifth percentile per tissue section were eliminated, as were genes detected in fewer than 10 spots per section. Our comprehensive LUAD scRNA‐seq dataset served as the reference when applying cell2location. The deconvolution was performed with default parameter settings, with adjustments to max_epochs = 12 000, batch_size = 15 000, n_cells_per_location = 30, and detection_alpha = 20. Resulting cell‐type abundance estimates were normalized to a 0–1 scale for each spot. Spots were assigned to the cell type with the dominant normalized abundance, provided its ratio relative to the second‐highest abundance surpassed the 10th percentile of the ratio distribution across all spots in the section.

### Identification of Tumor Borders in Spatial Transcriptomes

Following cell‐type assignment to spots in the spatial transcriptomics data, binary images of the tissue sections were created based on inferred AT2‐like cell concentrations. These images underwent thresholding and Gaussian blurring, followed by Canny edges detection. Regions enriched in AT2‐like cells, indicative of tumors, were delineated using OpenCV (Python version, v4.6.0), with contours of the regions refined through OpenCV functions of “cv2.approxPolyDP()” and “cv2.morphologyEx().” The contour encompassing the most extensive tumor region was designated as the region of interest. A border line was then established to distinguish tumor from adjacent normal tissue, and the surrounding zone was divided six 500‐µm‐wide bands by constructing parallel offsets extending 500, 1000, and 1500 µm perpendicularly on both sides, utilizing the “shapely.geometry.LineString.buffer()” function in Shapely (v2.0.0), https://shapely.readthedocs.io/en/stable/geometry.html). Each band included roughly 10 spots, which were selected for subsequent evaluation of cellular components and gene expression profiles.

### Subtypes Identification for AT2‐Like and Fibroblast Cells in Spatial Transcriptomic Data

After cell‐types annotations, we extracted spots assigned to AT2‐like or fibroblast cells from the spatial transcriptomics datasets. Mirroring the single‐cell RNA sequencing pipeline, gene expression per spot was normalized and log‐transformed. Z‐scores were derived for the top 1500 highly variable genes (HVGs), followed by principal component analysis (PCA) to isolate the primary principal components. Batch effects between samples were alleviated with harmonypy (0.0.6)^[^ ^60^ ^]^ with default parameters. We then conducted Leiden clustering with a resolution parameter of 0.3 to assign subtypes based on the known marker genes.^[^ ^13^ ^]^

A similar procedure was used to identify subtypes of fibroblasts, which were defined based on established marker genes profiles.^[^ ^46^, ^61^ ^]^ In detail, datasets from 15 tissue samples (spanning 16 219 spots and 12 351 genes) underwent clustering by fibroblast subtype via Scanpy. Spots exhibiting the lowest 10% purity within each sample were discarded. Upon dataset integration across the 15 samples, the top 1500 HVGs were identified using the mean‐to‐variance expression ratio and processed with log1p normalization. Principal component analysis (PCA) was carried out, retaining the top 20 principal components. Following batch correction via Harmony, Leiden clustering was applied at a resolution of 0.3. Every identified fibroblast subtypes displayed expression of hallmark fibroblast genes, such as ACTA2 and DCN.^[^ ^50^, ^62^ ^]^

### Copy Number Variation Analysis in AT2‐Like Subtypes from Spatial Transcriptomic Data

Copy number variations (CNVs) were inferred across AT2‐like subtypes using infercnvpy (v0.4.0). Genes were allocated to chromosomal loci according to the GRCh38 human reference genome, and a moving average of gene expression per subtype was computed via a 250‐gene sliding window. We used the scRNA‐seq data from AT2 cells in normal tissue as the reference baseline. The “infercnvpy.tl.infercnv()” function was applied with default parameters to estimate CNVs. Each spatial transcriptomics spot received a CNV score reflecting potential chromosomal gains or losses, where higher scores indicated an increased probability of CNV gain. To refine tumor cell annotations, we reclustered these cell populations based on the derived CNV scores.

### RNA Sequencing and Analysis

RNA extraction and library preparation: Total RNA was isolated using the RNeasy Mini Kit (Qiagen, Hilden, Germany). RNA integrity was verified using an Agilent 2100 Bioanalyzer (RIN ≥ 8.0). Strand‐specific mRNA libraries were constructed with the TruSeq Stranded mRNA Sample Prep Kit (Illumina, San Diego, CA), including poly‐A selection and dual‐index adaptor ligation. Sequencing and data processing: Libraries were sequenced on an Illumina NovaSeq 6000 platform (2 × 150 bp paired end; 30 M reads/sample). Raw reads underwent quality control (FastQC v0.11.9), adapter trimming (Trimmomatic v0.39), and alignment to the GRCh38 human reference genome using STAR v2.7.10a. Gene‐level counts were generated with featureCounts v2.0.3 (ENSEMBL v105 annotation). Differential expression analysis: Differential expression between NC and SLPI‐KD groups was assessed using DESeq2 v1.34.0 in R. Genes with |log_2_ (fold change) | > 1 and adjusted *p*‐value (Benjamini‐Hochberg) < 0.05 were considered significantly differentially expressed. Gene ontology enrichment was analyzed using clusterProfiler v4.2.2.

### Flow Cytometry for Lung RTM‐TAM Isolation and Analysis

Lung tissues from mice were minced, enzymatically digested (1 mg mL^−1^ collagenase IV + 0.02 mg mL^−1^ DNase I, 37 °C, 45 min), and filtered through 70‐µm strainers. Erythrocytes were lysed with ACK buffer, followed by leukocyte enrichment via 70% Percoll density gradient centrifugation. Cells were incubated with fluorochrome‐conjugated antibodies against L/D (BD, BV510), CD45 (BD; APC/Cy7), CD11b (BD, BV421), F4/80 (BD, FITC), and MRC1 (BD, PE). Fluorescence‐minus‐one (FMO) and isotype controls were included. After fixation in 2% PFA, 100 000 events/sample were acquired on a BD FACSAria II, with RTM‐TAMs identified as Live/CD45⁺/CD11b⁺/F4/80⁺/MRC1⁺ using FlowJo v10.8.1.

### Co‐Culture and Cytokine Analysis

Sorted lung RTM‐TAMs (Live/CD45⁺/CD11b⁺/F4/80⁺/MRC1⁺) were co‐cultured with SLPI‐overexpressing LLC cells (validated by qPCR/western blotting) at a 1:5 macrophage: tumor cell ratio in DMEM/10% FBS for 48 h at 37 °C. Supernatants were centrifuged (300 × g, 10 min) to remove debris and stored at −80 °C. Cytokine concentrations (IL‐12p70, IL‐10, IL‐6, TNF‐α, IFN‐γ, MCP‐1) were quantified using the Mouse Inflammation CBA Kit (Four Color Biotech, Beijing, China) according to the manufacturer's protocol. Briefly, 50 µL of supernatant was mixed with antibody‐conjugated beads, incubated for 2 h in the dark, washed, and acquired on a BD FACSVerse flow cytometer. Standard curves (0–5000 pg mL^−1^) and blank controls were included. Data analysis utilized FCAP Array v3.0 (BD Biosciences) with detection thresholds of 2.6 pg mL^−1^ (IL‐6) to 17.5 pg mL^−1^ (IL‐12p70).

### Identification and Analysis of Differentially Expressed Genes

Differentially expressed genes (DEGs) across subtypes of different cell types from MIA to IA tumors were identified via the “scanpy.tl.rank_genes_groups()” function in Scanpy. Genes were defined as differentially expressed if |log_2_ (fold change) | ≥ 1.5 and an adjusted p < 0.05 based on the t‐test with Benjamini‐Hochberg correction.

The biological roles of these DEGs were assessed through enrichment analysis in GO terms and KEGG pathways, employing the cluster Profiler package (4.6.0) in R (v4.2.2). Significant enrichment was determined by an adjusted p‐value < 0.05 following false discovery rate (FDR) correction.

### Trajectory Inference Analysis in AT2 Cells and AT2‐Like Subtypes from Spatial Transcriptomics Data

Cellular trajectories and pseudotime were inferred for AT2 cells and AT2‐like subtypes in spatially resolved transcriptomics (SRT) data using Monocle3 (version 1.3.1),^[^ ^29^, ^30^, ^63^, ^64^ ^]^ following the official tutorial (https://cole‐trapnell‐lab.github.io/monocle3/docs/trajectories/). Specifically, Filtered SRT data, restricted to high‐quality spots annotated as AT2 cells or AT2‐like subtypes, were imported into a Monocle3 cell_data_set (CDS) object using the “new_cell_data_set()” function. This included parameters for cell_metadata (incorporating spot annotations and a “batch” variable for tissue slide IDs) and gene_metadata (specifying gene names). Data preprocessing was performed using the “preprocess_cds()” function under default settings (method = “PCA”, num_dim = 50, norm_method = “log”) to normalize expression values and reduce dimensionality. Batch effects across tissue slides were mitigated via the “align_cds()” function with default parameters. Further dimensionality reduction was applied using uniform UMAP through the “reduce_dimension()” function (default parameters). Spots within the CDS were clustered with the Leiden algorithm via the “cluster_cells()” function (default parameters), grouping them based on similar expression profiles. Trajectory inference was performed by learning a principal graph over the reduced‐dimensional space using the “learn_graph()” function (default parameters). Pseudotime was assigned with the “order_cells()” function, projecting spots onto the trajectory backbone for ordering. Trajectories were rooted at AT2 cells, selected as starting points based on biological priors. Genes varying significantly along pseudotime were identified through differential expression analysis with the “graph_test()” function, employing Moran's I statistic (*q*‐value < 0.05). Outputs were visualized as UMAP plots colored by pseudotime and heatmaps of differentially expressed genes ordered by pseudotime.

### Gene‐Set Scoring and Variation Analysis

The “scanpy.tl.score_genes()” function in Scanpy (v1.9.1) was employed to calculate gene‐set score, derived from the average expression levels of differentially expressed genes or marker genes from the literature.^[^ ^13^, ^35^, ^65^ ^]^ Furthermore, GSVA was applied to compute scores for tumor hallmark gene expression, as curated in the Molecular Signature Database (MSigDB, v1.46.0).^[^ ^66^ ^]^

### Cell‐Cell Communication Analysis​

We deduced intercellular communication networks using the CellChat^[^ ^67^ ^]^ package (v1.6.1). Briefly, a CellChat object was generated by importing the normalized gene expression matrix and associated cell type labels. The analysis drew upon the built‐in human ligand‐receptor interaction database (CellChatDB.human, v1). Cell‐type‐specific communications were determined by first detecting overexpressed ligands or receptors in individual cell populations, followed by pinpointing amplified ligand–receptor pairs where either component showed overexpression. The communication probabilities across cell populations were calculated via the “computeCommunProb()” function with default parameter settings. Significant ligand‐receptor pairs were then visualized using the “netVisual_bubble()” function, applying the default significance threshold (*p* < 0.05) to retain only statistically robust interactions. In the generated bubble plot, dot color indicated the average communication probability for each pair, while dot size reflected statistical significance represented by p‐value. Interactions were excluded if any involved cell population contained fewer than 10 cells.

### Effects of SLPI Knockdown in Cell Culture

PC9 cells (Cell Bank of the Chinese Academy of Sciences, Shanghai, China) were cultured in RPMI‐1640 supplemented with 10% FBS (37 °C, 5% CO_2_). Cells were transfected with a short hairpin RNA targeting SLPI (5′‐ GAGTCTGTCCTCCTAAGAAAT‐3′) or a negative‐control short hairpin RNA (5′‐TTCTCCGAACGTGTCACGT‐3′) using Lipofectamine 3000 (Invitrogen, CA, USA). RNAs were synthesized by GeneChem (Shanghai, China). Transfectants were selected on the basis of their expression of green fluorescent protein.

Effects of SLPI knockdown on cancer cell migration were evaluated via a wound‐healing assay. Cells were incubated for 24 h in six‐well plates (2 × 10^5^ cells per well), after which the cell monolayer was scratched using a sterile pipette. Cells were washed with phosphate‐buffered saline and cultured for 48 h, at the start and end of which cultures were photographed under an inverted microscope.

The impact of SLPI knockdown on cancer cell invasion was assessed using Transwell chambers with 8‐µm pores (Corning, NY, USA) precoated with Matrigel (Corning). Cells suspended in serum‐free RPMI‐1640 medium were plated in the upper compartment, while the lower compartment received medium supplemented with 10% FBS. Plates were incubated for 24 h, cells on the lower membrane surface were fixed in methanol for 15 min, stained using 0.1% crystal violet, and enumerated.

Effects of SLPI knockdown on cancer cell proliferation were evaluated using the Cell Counting Kit‐8 assay (Yeasen, Shanghai, China). Cells were seeded into 96‐well plates (4 × 10^3^ cells per well), cultured for 24–96 h, and then assayed with the kit.

Effects of SLPI knockdown on expression of proteins involved in cancer progression and metastasis were assessed by western blotting of total lysates from cells expressing endogenous or knocked‐down levels of SLPI. Cells were rinsed twice with PBS, lysed in lysis buffer, and centrifuged (13 000×g, 15 min). The supernatant was used to determine total protein concentration via an enhanced bicinchoninic acid assay (Biosharp, Hefei, China); equal amounts of protein were then separated by 10% sodium dodecyl sulfate‐polyacrylamide gel electrophoresis (SDS‐PAGE). Proteins were transferred to nitrocellulose membranes, which were blocked with 5% skim milk and subsequently incubated with appropriate primary antibodies, including CD133 (CST, #64 326), SOX2 (CST, #4900), DKK1 (Affinity, #AF4600), beta‐catenin (Affinity, #AF6266), Cyclin D1 (Affinity, #0931), N‐Cadherin (CST, #13 116), E‐Cadherin (CST, #14 472), Snail (CST, #9585). Membranes were rinsed twice with TBS‐T and then incubated with appropriate secondary antibodies linked to horseradish peroxidase. Antibody binding was visualized via enhanced chemiluminescence (4A biotech, 4AW011‐1000) and quantitated using Image J.

Effects of SLPI knockdown on growth of LUAD tumors were evaluated by injecting PC9 cells expressing endogenous levels or knocked‐down levels of SLPI subcutaneously into male BALB/c mice (6–8 weeks, 20–22 g, 2 × 10^6^ cells per mouse; GemPharmatech, Nanjing, China). Body weight and tumor volume V = (L × W^2^) / 2 were measured every 3 days. On 21 days after injection of cancer cells, animals were sacrificed, and xenografts were excised for analysis. In separate experiments to assess effects of SLPI knockdown on metastasis of LUAD cells, PC9 cells expressing endogenous or knocked‐down levels of SLPI were injected intravenously into mice (2 × 10^6^ cells per mouse). After 40 days, animals were sacrificed and lung tissues were collected, photographed, and fixed in 4% paraformaldehyde for further analysis. All animal experimental procedures received approval from the Animal Ethical and Welfare Committee of West China Hospital of Sichuan University.

Immunohistochemistry (IHC) was performed with formalin‐fixed paraffin‐embedded (FFPE) tissue using standard protocols. Isolated tumor tissues were immediately fixed with 4% paraformaldehyde overnight. Paraffin‐embedded tissues were cut at 4 µm and stained for IHC. The slides were submerged into citrate buffer (pH 6.0) for heat‐induced antigen retrieval. Then, these slides were incubated with goat serum and incubated with the following primary antibodies at 4 °C overnight: Ki67 (Cell Signaling, #12 202, 1:500), SLPI (Bioss, bs‐6849R, 1:500). The slides were then washed, and secondary antibody was used for further incubation. Diaminobenzidine (Zhongshan Golden Bridge, Beijing, China) was used for color development. Then these slides were counterstained with hematoxylin and dehydrated.

### Statistical Analysis

Statistical evaluations were conducted using Student's t‐test, Wilcoxon signed‐rank test, and Wilcoxon rank‐sum test, as appropriate for the data distribution and comparisons. Differences were regarded as statistically significant at *p*‐values < 0.05 across all analyses. Significance levels are denoted by asterisks in figures (* *p* < 0.05, ** *p* < 0.01, *** *p* < 0.001, **** *p* < 0.0001; ns, not significant). In boxplots, boxes represent the interquartile range (IQR; 25th–75th percentiles) with the central line indicating the median, and whiskers extending to 1.5×IQR.

### Data and Materials Availability

The generated WES, WGS and RNA‐seq data in this study have been deposited to Genome Sequence Archive (GSA) in BIG Data Center, Beijing Institute of Genomics (BIG) under accession number HRA009069 (https://ngdc.cncb.ac.cn/gsa‐human/browse/HRA009069).

## Conflict of Interest

The authors declare no conflict of interest.

## Author Contributions

Z.W., G.Z., P.T., and Y.W. contributed equally to this work. Conceptualization: W.M.L., Y.B., and Z.F.W. Methodology: Z.F.W., G.N.Z., and P.T. Investigation: Y.W., W.X.L., W.P.S., Z.K.P., B.J.Z., Y.C.J., D.F.X., and X.J. Supervision: G.W.C. Writing‐original draft: Z.F.W. and Y.B. Writing‐review & editing: W.M.L.

## Supporting information



Supporting Information

## Data Availability

The data that support the findings of this study are available from the corresponding author upon reasonable request.

## References

[1] H. Sung , J. Ferlay , R. L. Siegel , M. Laversanne , I. Soerjomataram , A. Jemal , F. Bray , CA Cancer J. Clin. 2021, 71, 209. 33538338 10.3322/caac.21660

[2] J. Lortet‐Tieulent , I. Soerjomataram , J. Ferlay , M. Rutherford , E. Weiderpass , F. Bray , Lung Cancer 2014, 84, 13. 24524818 10.1016/j.lungcan.2014.01.009

[3] S. Wang , M. Du , J. Zhang , W. Xu , Q. Yuan , M. Li , J. Wang , H. Zhu , Y. Wang , C. Wang , Y. Gong , X. Wang , Z. Hu , D. C. Christiani , L. Xu , H. Shen , R. Yin , Nat. Commun. 2020, 11, 6083. 33247113 10.1038/s41467-020-19855-x PMC7695730

[4] G. D. Jones , W. S. Brandt , R. L. Shen , F. Sanchez‐Vega , K. S. Tan , A. Martin , J. Zhou , M. Berger , D. B. Solit , N. Schultz , H. Rizvi , Y. Liu , A. Adamski , J. E. Chaft , G. J. Riely , G. Rocco , M. J. Bott , D. Molena , M. Ladanyi , W. D. Travis , N. Rekhtman , B. J. Park , P. S. Adusumilli , D. Lyden , M. Imielinski , M. W. Mayo , B. T. Li , D. R. A. Jones , Jama Surg. 2021, 156, 205601. 10.1001/jamasurg.2020.5601 PMC7758824 33355651

[5] S. Xu , J. Zhou , K. Liu , Z. Chen , Z. He , Biomed Res. Int. 2020, 2020, 9124792. 33224985 10.1155/2020/9124792 PMC7669350

[6] Z. Yang , J. Luo , M. Zhang , M. Zhan , Y. Bai , Y. Yang , W. Wang , L. Lu , Heliyon 2023, 9, 21505. 10.1016/j.heliyon.2023.e21505 PMC10663839 38027718

[7] J. G. Edwards , K. Chansky , P. Van Schil , A. G. Nicholson , S. Boubia , E. Brambilla , J. Donington , F. Galateau‐Salle , H. Hoffmann , M. Infante , M. Marino , E. M. Marom , J. Nakajima , M. Ostrowski , W. D. Travis , M. S. Tsao , Y. Yatabe , D. J. Giroux , L. Shemanski , J. Crowley , M. Krasnik , H. Asamura , J. Thorac. Oncol. 2020, 15, 344.31731014

[8] Z. Chen , C. M. Fillmore , P. S. Hammerman , C. F. Kim , K. K. Wong , Nat. Rev. Cancer 2014, 14, 535. 25056707 10.1038/nrc3775 PMC5712844

[9] Y. Goltsev , N. Samusik , J. Kennedy‐Darling , S. Bhate , M. Hale , G. Vazquez , S. Black , G. P. Nolan , Cell 2018, 174, 968. 30078711 10.1016/j.cell.2018.07.010 PMC6086938

[10] Y. Salazar , X. Zheng , D. Brunn , H. Raifer , F. Picard , Y. Zhang , H. Winter , S. Guenther , A. Weigert , B. Weigmann , L. Dumoutier , J. C. Renauld , A. Waisman , A. Schmall , A. Tufman , L. Fink , B. Brune , T. Bopp , F. Grimminger , W. Seeger , S. S. Pullamsetti , M. Huber , R. Savai , J. Clin. Invest. 2020, 130, 3560. 32229721 10.1172/JCI124037 PMC7324180

[11] N. Kim , H. K. Kim , K. Lee , Y. Hong , J. H. Cho , J. W. Choi , J. I. Lee , Y. L. Suh , B. M. Ku , H. H. Eum , S. Choi , Y. L. Choi , J. G. Joung , W. Y. Park , H. A. Jung , J. M. Sun , S. H. Lee , J. S. Ahn , K. Park , M. J. Ahn , L. HO , Nat. Commun. 2020, 11, 2285. 32385277 10.1038/s41467-020-16164-1 PMC7210975

[12] F. Wu , J. Fan , Y. He , A. Xiong , J. Yu , Y. Li , Y. Zhang , W. Zhao , F. Zhou , W. Li , J. Zhang , X. Zhang , M. Qiao , G. Gao , S. Chen , X. Chen , X. Li , L. Hou , C. Wu , C. Su , S. Ren , M. Odenthal , R. Buettner , N. Fang , C. Zhou , Nat. Commun. 2021, 12, 2540. 33953163 10.1038/s41467-021-22801-0 PMC8100173

[13] Z. Wang , Z. Li , K. Zhou , C. Wang , L. Jiang , L. Zhang , Y. Yang , W. Luo , W. Qiao , G. Wang , Y. Ni , S. Dai , T. Guo , G. Ji , M. Xu , Y. Liu , Z. Su , G. Che , W. Li , Nat. Commun. 2021, 12, 6500. 34764257 10.1038/s41467-021-26770-2 PMC8586023

[14] W. Luo , Z. Zeng , Y. Jin , L. Yang , T. Fan , Z. Wang , Y. Pan , Y. Yang , M. Yao , Y. Li , X. Xiao , G. Wang , C. Wang , S. Chang , G. Che , L. Zhang , Y. Li , Y. Peng , W. Li , Cell Rep. Med. 2023, 4, 101078. 37301197 10.1016/j.xcrm.2023.101078 PMC10313938

[15] L. Zhang , Y. Zhang , C. Wang , Y. Yang , Y. Ni , Z. Wang , T. Song , M. Yao , Z. Liu , N. Chao , Y. Yang , J. Shao , Z. Li , R. Zhou , L. Chen , D. Zhang , Y. Zhao , W. Liu , Y. Li , P. He , J. W. Lin , Y. Wang , K. Zhang , L. Chen , W. Li , Signal Transduction Targeted Ther. 2022, 7, 9. 10.1038/s41392-021-00824-9 PMC8758688 35027529

[16] V. Marx , Nat. Methods 2021, 18, 9. 34413524 10.1038/s41592-021-01258-5

[17] E. Denisenko , L. de Kock , A. Tan , A. B. Beasley , M. Beilin , M. E. Jones , R. Hou , D. O. Muiri , S. Bilic , G. Mohan , S. Salfinger , S. Fox , K. P. W. Hmon , Y. Yeow , Y. Kim , R. John , T. S. Gilderman , E. Killingbeck , E. S. Gray , P. A. Cohen , Y. Yu , A. R. R. Forrest , Nat. Commun. 2024, 15, 2860. 38570491 10.1038/s41467-024-47271-y PMC10991508

[18] A. M. Leader , J. A. Grout , B. B. Maier , B. Y. Nabet , M. D. Park , A. Tabachnikova , C. Chang , L. Walker , A. Lansky , J. Le Berichel , L. Troncoso , N. Malissen , M. Davila , J. C. Martin , G. Magri , K. Tuballes , Z. Zhao , F. Petralia , R. Samstein , N. R. D'Amore , G. Thurston , A. O. Kamphorst , A. Wolf , R. Flores , P. Wang , S. Muller , I. Mellman , M. B. Beasley , H. Salmon , A. H. Rahman , et al., Cancer Cell 2021, 39, 1594. 34767762 10.1016/j.ccell.2021.10.009 PMC8728963

[19] D. A. Lawson , K. Kessenbrock , R. T. Davis , N. Pervolarakis , Z. Werb , Nat. Cell Biol. 2018, 20, 1349. 30482943 10.1038/s41556-018-0236-7 PMC6477686

[20] A. Andersson , J. Bergenstrahle , M. Asp , L. Bergenstrahle , A. Jurek , J. Fernandez Navarro , J. Lundeberg , Commun. Biol. 2020, 3, 565. 33037292 10.1038/s42003-020-01247-y PMC7547664

[21] A. L. Ji , A. J. Rubin , K. Thrane , S. Jiang , D. L. Reynolds , R. M. Meyers , M. G. Guo , B. M. George , A. Mollbrink , J. Bergenstrahle , L. Larsson , Y. Bai , B. Zhu , A. Bhaduri , J. M. Meyers , X. Rovira‐Clave , S. T. Hollmig , S. Z. Aasi , G. P. Nolan , J. Lundeberg , P. A. Khavari , Cell 2020, 182, 497. 32579974 10.1016/j.cell.2020.05.039 PMC7391009

[22] P. L. Stahl , F. Salmen , S. Vickovic , A. Lundmark , J. F. Navarro , J. Magnusson , S. Giacomello , M. Asp , J. O. Westholm , M. Huss , A. Mollbrink , S. Linnarsson , S. Codeluppi , A. Borg , F. Ponten , P. I. Costea , P. Sahlen , J. Mulder , O. Bergmann , J. Lundeberg , J. Frisen , Science 2016, 353, 78. 27365449 10.1126/science.aaf2403

[23] L. Wu , J. Yan , Y. Bai , F. Chen , X. Zou , J. Xu , A. Huang , L. Hou , Y. Zhong , Z. Jing , Q. Yu , X. Zhou , Z. Jiang , C. Wang , M. Cheng , Y. Ji , Y. Hou , R. Luo , Q. Li , L. Wu , J. Cheng , P. Wang , D. Guo , W. Huang , J. Lei , S. Liu , Y. Yan , Y. Chen , S. Liao , Y. Li , et al., Cell Res. 2023, 33, 585. 37337030 10.1038/s41422-023-00831-1 PMC10397313

[24] Z. Ou , S. Lin , J. Qiu , W. Ding , P. Ren , D. Chen , J. Wang , Y. Tong , D. Wu , A. Chen , Y. Deng , M. Cheng , T. Peng , H. Lu , H. Yang , J. Wang , X. Jin , D. Ma , X. Xu , Y. Wang , J. Li , P. Wu , Adv. Sci. (Weinh) 2022, 9, 2203040. 35986392 10.1002/advs.202203040 PMC9561780

[25] F. A. Wolf , P. Angerer , F. J. Theis , Genome Biol. 2018, 19, 15. 29409532 10.1186/s13059-017-1382-0 PMC5802054

[26] C. Wang , Q. Yu , T. Song , Z. Wang , L. Song , Y. Yang , J. Shao , J. Li , Y. Ni , N. Chao , L. Zhang , W. Li , Signal Transduction Targeted Ther. 2022, 7, 289. 10.1038/s41392-022-01130-8 PMC9411197 36008393

[27] K. Curtius , N. A. Wright , T. A. Graham , Nat. Rev. Cancer 2018, 18, 19. 29217838 10.1038/nrc.2017.102

[28] P. R. Tata , H. Mou , A. Pardo‐Saganta , R. Zhao , M. Prabhu , B. M. Law , V. Vinarsky , J. L. Cho , S. Breton , A. Sahay , B. D. Medoff , J. Rajagopal , Nature 2013, 503, 218. 24196716 10.1038/nature12777 PMC4035230

[29] X. Qiu , A. Hill , J. Packer , D. Lin , Y. A. Ma , C. Trapnell , Nat. Methods 2017, 14, 309. 28114287 10.1038/nmeth.4150 PMC5330805

[30] X. Qiu , Q. Mao , Y. Tang , L. Wang , R. Chawla , H. A. Pliner , C. Trapnell , Nat. Methods 2017, 14, 979. 28825705 10.1038/nmeth.4402 PMC5764547

[31] Q. Chen , H. Hirai , M. Chan , J. Zhang , M. Cho , S. H. Randell , P. Kadur Lakshminarasimha Murthy , J. Rehman , Y. Liu , Stem Cell Rep. 2024, 19, 890. 10.1016/j.stemcr.2024.04.009 PMC11390684 38759645

[32] S. A. Toulmin , C. Bhadiadra , A. J. Paris , J. H. Lin , J. Katzen , M. C. Basil , E. E. Morrisey , G. S. Worthen , L. C. Eisenlohr , Nat. Commun. 2021, 12, 3993. 34183650 10.1038/s41467-021-23619-6 PMC8239023

[33] B. A. Otalora‐Otalora , D. A. Osuna‐Garzon , M. S. Carvajal‐Parra , A. Canas , M. Montecino , L. Lopez‐Kleine , A. Rojas , Biology (Basel) 2022, 11, 1082. 36101460 10.3390/biology11071082 PMC9313083

[34] L. Li , L. L. Wang , T. L. Wang , F. M. Zheng , Med. Oncol. 2023, 40, 118. 36929466 10.1007/s12032-023-01978-y

[35] C. Riganti , E. Gazzano , M. Polimeni , E. Aldieri , D. Ghigo , Free Radic. Biol. Med. 2012, 53, 421. 22580150 10.1016/j.freeradbiomed.2012.05.006

[36] A. Erickson , M. He , E. Berglund , M. Marklund , R. Mirzazadeh , N. Schultz , L. Kvastad , A. Andersson , L. Bergenstrahle , J. Bergenstrahle , L. Larsson , L. Alonso Galicia , A. Shamikh , E. Basmaci , T. Diaz De Stahl , T. Rajakumar , D. Doultsinos , K. Thrane , A. L. Ji , P. A. Khavari , F. Tarish , A. Tanoglidi , J. Maaskola , R. Colling , T. Mirtti , F. C. Hamdy , D. J. Woodcock , T. Helleday , I. G. Mills , A. D. Lamb , et al., Nature 2022, 608, 360. 35948708 10.1038/s41586-022-05023-2 PMC9365699

[37] E. Wagenblast , M. Soto , S. Gutierrez‐Angel , C. A. Hartl , A. L. Gable , A. R. Maceli , N. Erard , A. M. Williams , S. Y. Kim , S. Dickopf , J. C. Harrell , A. D. Smith , C. M. Perou , J. E. Wilkinson , G. J. Hannon , S. R. Knott , Nature 2015, 520, 358. 25855289 10.1038/nature14403 PMC4634366

[38] R. Y. Ma , A. Black , B. Z. Qian , Trends Immunol. 2022, 43, 546. 35690521 10.1016/j.it.2022.04.008

[39] X. Liu , Y. Cai , C. Cheng , Y. Gu , X. Hu , K. Chen , Y. Wu , Z. Wu , Cell Death Dis. 2022, 13, 178. 35210406 10.1038/s41419-022-04635-w PMC8873510

[40] J. Q. Luo , T. W. Yang , J. Wu , H. H. Lai , L. B. Zou , W. B. Chen , X. M. Zhou , D. J. Lv , S. R. Cen , Z. N. Long , Y. Y. Mao , P. X. Zheng , X. H. Su , Z. Y. Xian , F. P. Shu , X. M. Mao , Cell Death Dis. 2023, 14, 502. 37542027 10.1038/s41419-023-06007-4 PMC10403531

[41] M. Casanova‐Acebes , E. Dalla , A. M. Leader , J. LeBerichel , J. Nikolic , B. M. Morales , M. Brown , C. Chang , L. Troncoso , S. T. Chen , A. Sastre‐Perona , M. D. Park , A. Tabachnikova , M. Dhainaut , P. Hamon , B. Maier , C. M. Sawai , E. Agullo‐Pascual , M. Schober , B. D. Brown , B. Reizis , T. Marron , E. Kenigsberg , C. Moussion , P. Benaroch , J. A. Aguirre‐Ghiso , M. Merad , Nature 2021, 595, 578. 34135508 10.1038/s41586-021-03651-8 PMC8923521

[42] Z. Wu , C. Huang , Int. J. Gen. Med. 2024, 17, 2575. 38855425 10.2147/IJGM.S461144 PMC11162242

[43] A. D. Donnenberg , L. Zimmerlin , R. J. Landreneau , J. D. Luketich , V. S. Donnenberg , PLoS One 2012, 7, 52885. 10.1371/journal.pone.0052885 PMC3527622 23285214

[44] D. Yang , J. Liu , H. Qian , Q. Zhuang , Exp. Mol. Med. 2023, 55, 1322. 37394578 10.1038/s12276-023-01013-0 PMC10394065

[45] H. Su , N. Na , X. Zhang , Y. Zhao , Inflamm Res. 2017, 66, 209. 27752708 10.1007/s00011-016-0995-1

[46] M. Tsoumakidou , Nat. Rev. Cancer 2023, 23, 258. 36807417 10.1038/s41568-023-00549-7

[47] E. Dantas , A. Murthy , T. Ahmed , M. Ahmed , S. Ramsamooj , M. A. Hurd , T. Lam , M. Malbari , C. Agrusa , O. Elemento , C. Zhang , D. J. Pappin , T. E. McGraw , B. M. Stiles , N. K. Altorki , M. D. Goncalves , Clin. Transl. Med. 2023, 13, 1391. 10.1002/ctm2.1391 PMC10533479 37759102

[48] Y. Chen , J. Kim , S. Yang , H. Wang , C. J. Wu , H. Sugimoto , V. S. LeBleu , R. Kalluri , Cancer Cell 2021, 39, 548. 33667385 10.1016/j.ccell.2021.02.007 PMC8423173

[49] A. N. Hosein , H. Huang , Z. Wang , K. Parmar , W. Du , J. Huang , A. Maitra , E. Olson , U. Verma , R. A. Brekken , JCI Insight 2019, 5. 10.1172/jci.insight.129212 PMC6777805 31335328

[50] D. Ohlund , A. Handly‐Santana , G. Biffi , E. Elyada , A. S. Almeida , M. Ponz‐Sarvise , V. Corbo , T. E. Oni , S. A. Hearn , E. J. Lee , I. I. Chio , C. I. Hwang , H. Tiriac , L. A. Baker , D. D. Engle , C. Feig , A. Kultti , M. Egeblad , D. T. Fearon , J. M. Crawford , H. Clevers , Y. Park , D. A. Tuveson , J. Exp. Med. 2017, 214, 579. 28232471 10.1084/jem.20162024 PMC5339682

[51] Q. Ji , L. Zhou , H. Sui , L. Yang , X. Wu , Q. Song , R. Jia , R. Li , J. Sun , Z. Wang , N. Liu , Y. Feng , X. Sun , G. Cai , Y. Feng , J. Cai , Y. Cao , G. Cai , Y. Wang , Q. Li , Nat. Commun. 2020, 11, 1211. 32139701 10.1038/s41467-020-14869-x PMC7058049

[52] H. Li , Y. Wang , S. K. Rong , L. Li , T. Chen , Y. Y. Fan , Y. F. Wang , C. R. Yang , C. Yang , W. C. Cho , J. Yang , Int. J. Biol. Sci. 2020, 16, 815. 32071551 10.7150/ijbs.37275 PMC7019142

[53] H. Yang , Y. Liu , M. M. Zhao , Q. Guo , X. K. Zheng , D. Liu , K. W. Zeng , P. F. Tu , Cell Death Dis. 2021, 12, 492. 33990545 10.1038/s41419-021-03780-y PMC8121893

[54] E. Sahai , I. Astsaturov , E. Cukierman , D. G. DeNardo , M. Egeblad , R. M. Evans , D. Fearon , F. R. Greten , S. R. Hingorani , T. Hunter , R. O. Hynes , R. K. Jain , T. Janowitz , C. Jorgensen , A. C. Kimmelman , M. G. Kolonin , R. G. Maki , R. S. Powers , E. Pure , D. C. Ramirez , R. Scherz‐Shouval , M. H. Sherman , S. Stewart , T. D. Tlsty , D. A. Tuveson , F. M. Watt , V. Weaver , A. T. Weeraratna , Z. Werb , Nat. Rev. Cancer 2020, 20, 174. 31980749 10.1038/s41568-019-0238-1 PMC7046529

[55] S. Hu , H. Lu , W. Xie , D. Wang , Z. Shan , X. Xing , X. M. Wang , J. Fang , W. Dong , W. Dai , J. Guo , Y. Zhang , S. Wen , X. Y. Guo , Q. Chen , F. Bai , Z. Wang , J. Clin. Invest. 2022, 132, 157649. 10.1172/JCI157649 PMC9525123 35972800

[56] Y. Kieffer , H. R. Hocine , G. Gentric , F. Pelon , C. Bernard , B. Bourachot , S. Lameiras , L. Albergante , C. Bonneau , A. Guyard , K. Tarte , A. Zinovyev , S. Baulande , G. Zalcman , A. Vincent‐Salomon , F. Mechta‐Grigoriou , Cancer Discovery 2020, 10, 1330. 32434947 10.1158/2159-8290.CD-19-1384

[57] D. Tavernari , E. Battistello , E. Dheilly , A. S. Petruzzella , M. Mina , J. Sordet‐Dessimoz , S. Peters , T. Krueger , D. Gfeller , N. Riggi , E. Oricchio , I. Letovanec , G. Ciriello , Cancer Discovery 2021, 11, 1490. 33563664 10.1158/2159-8290.CD-20-1274

[58] K. Chansky , F. C. Detterbeck , A. G. Nicholson , V. W. Rusch , E. Vallieres , P. Groome , C. Kennedy , M. Krasnik , M. Peake , L. Shemanski , V. Bolejack , J. J. Crowley , H. Asamura , R. Rami‐Porta , I. Staging , J. Thorac. Oncol. 2017, 12, 1109.28461257

[59] V. Kleshchevnikov , A. Shmatko , E. Dann , A. Aivazidis , H. W. King , T. Li , R. Elmentaite , A. Lomakin , V. Kedlian , A. Gayoso , M. S. Jain , J. S. Park , L. Ramona , E. Tuck , A. Arutyunyan , R. Vento‐Tormo , M. Gerstung , L. James , O. Stegle , O. A. Bayraktar , Nat. Biotechnol. 2022, 40, 661. 35027729 10.1038/s41587-021-01139-4

[60] I. Korsunsky , N. Millard , J. Fan , K. Slowikowski , F. Zhang , K. Wei , Y. Baglaenko , M. Brenner , P. R. Loh , S. Raychaudhuri , Nat Methods 2019, 16, 1289. 31740819 10.1038/s41592-019-0619-0 PMC6884693

[61] E. Elyada , M. Bolisetty , P. Laise , W. F. Flynn , E. T. Courtois , R. A. Burkhart , J. A. Teinor , P. Belleau , G. Biffi , M. S. Lucito , S. Sivajothi , T. D. Armstrong , D. D. Engle , K. H. Yu , Y. Hao , C. L. Wolfgang , Y. Park , J. Preall , E. M. Jaffee , A. Califano , P. Robson , D. A. Tuveson , Cancer Discovery 2019, 9, 1102. 31197017 10.1158/2159-8290.CD-19-0094 PMC6727976

[62] D. Lavie , A. Ben‐Shmuel , N. Erez , R. Scherz‐Shouval , Jul 2022, 3, 793. 10.1038/s43018-022-00411-z PMC7613625 35883004

[63] C. Trapnell , D. Cacchiarelli , J. Grimsby , P. Pokharel , S. Li , M. Morse , N. J. Lennon , K. J. Livak , T. S. Mikkelsen , J. L. Rinn , Nat. Biotechnol. 2014, 32, 381. 24658644 10.1038/nbt.2859 PMC4122333

[64] J. Cao , M. Spielmann , X. Qiu , X. Huang , D. M. Ibrahim , A. J. Hill , F. Zhang , S. Mundlos , L. Christiansen , F. J. Steemers , C. Trapnell , J. Shendure , Nature 2019, 566, 496. 30787437 10.1038/s41586-019-0969-x PMC6434952

[65] T. Tammela , F. J. Sanchez‐Rivera , N. M. Cetinbas , K. Wu , N. S. Joshi , K. Helenius , Y. Park , R. Azimi , N. R. Kerper , R. A. Wesselhoeft , X. Gu , L. Schmidt , M. Cornwall‐Brady , O. H. Yilmaz , W. Xue , P. Katajisto , A. Bhutkar , T. Jacks , Nature 2017, 545, 355. 28489818 10.1038/nature22334 PMC5903678

[66] S. Hanzelmann , R. Castelo , J. Guinney , BMC Bioinformatics 2013, 14, 7. 23323831 10.1186/1471-2105-14-7 PMC3618321

[67] S. Jin , C. F. Guerrero‐Juarez , L. Zhang , I. Chang , R. Ramos , C. H. Kuan , P. Myung , M. V. Plikus , Q. Nie , Nat. Commun. 2021, 12, 1088. 33597522 10.1038/s41467-021-21246-9 PMC7889871

