# Supplementary material for: SLPI⁺ AT2‐Like Cells Orchestrate Lung Adenocarcinoma Invasion via Wnt Pathway Activation and Stromal Crosstalk in a Spatially Defined Margin Niche
Source: Adv Sci (Weinh). 2025 Nov 11;13(6):e16580. doi: 10.1002/advs.202516580 (PMC12866786; doi:10.1002/advs.202516580)
Supplement: Supplementary file 1 — Supporting Information [file ADVS-13-e16580-s001.docx]

**Supporting Information**


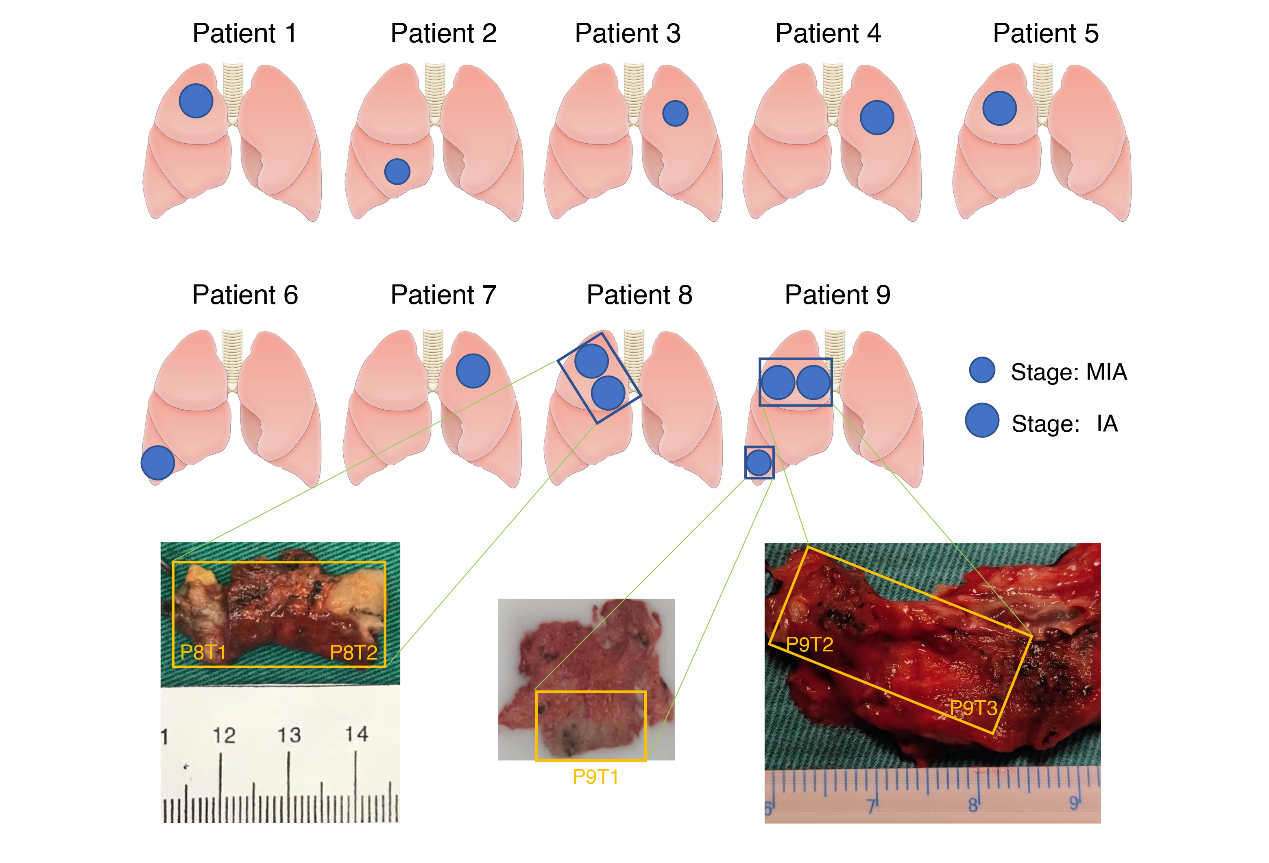


**Supplementary Figure 1.** **Clinical characteristics of LUAD patients enrolled in this study**. Nine patients with lung adenocarcinoma (LUAD) were included, comprising those with minimally invasive adenocarcinoma (MIA) and invasive adenocarcinoma (IA). Patients 8 and 9 presented with multifocal tumors.


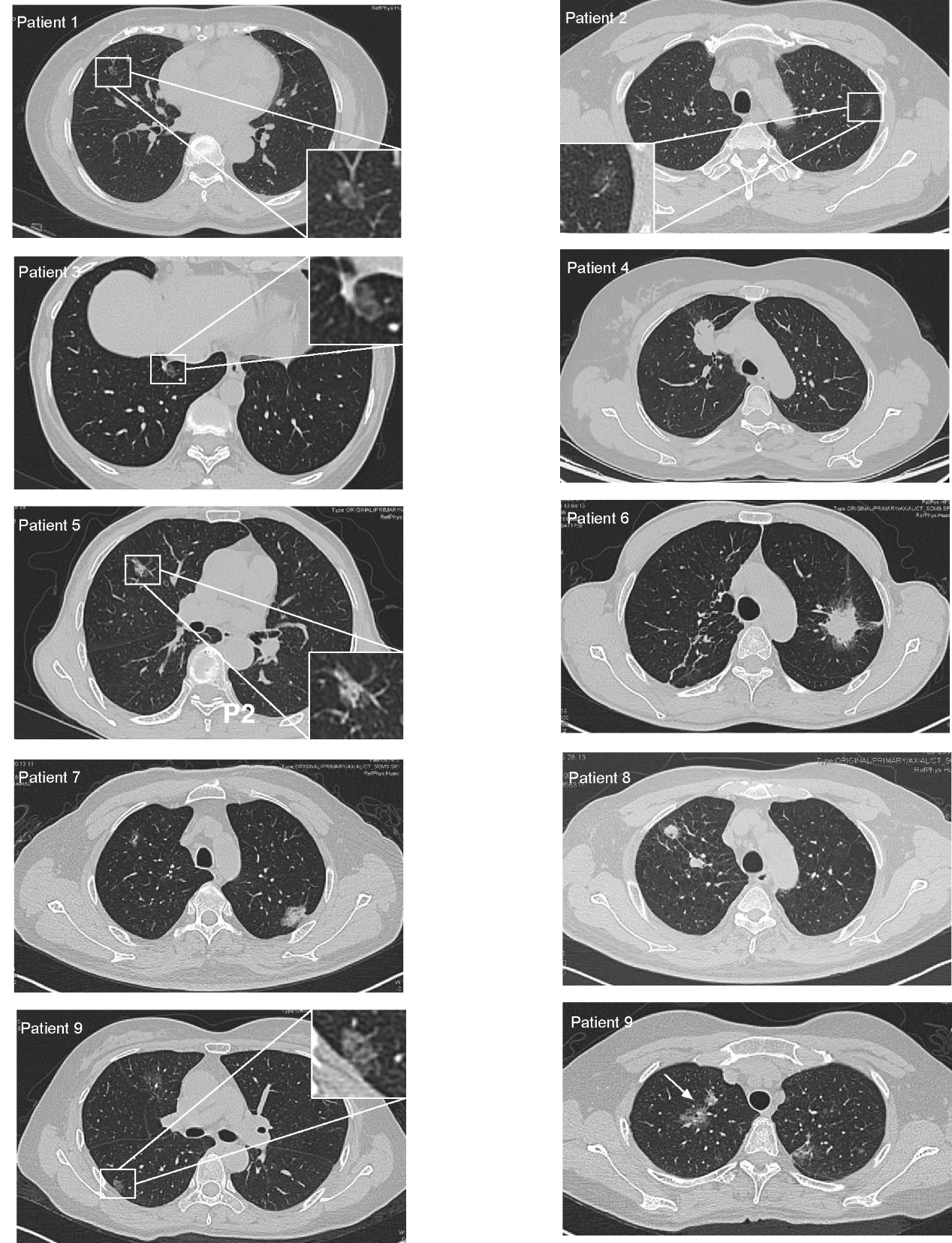


**Supplementary Figure 2. Computed tomography imaging LUAD lesions.** Representative CT scan images of lung tissue from the indicated patients. Tumor lesions are highlighted with boxes, shown at higher magnification in insects, or indicated with arrows. Resected tumor specimens from these nine patients were subjected to Stereo-seq analysis.


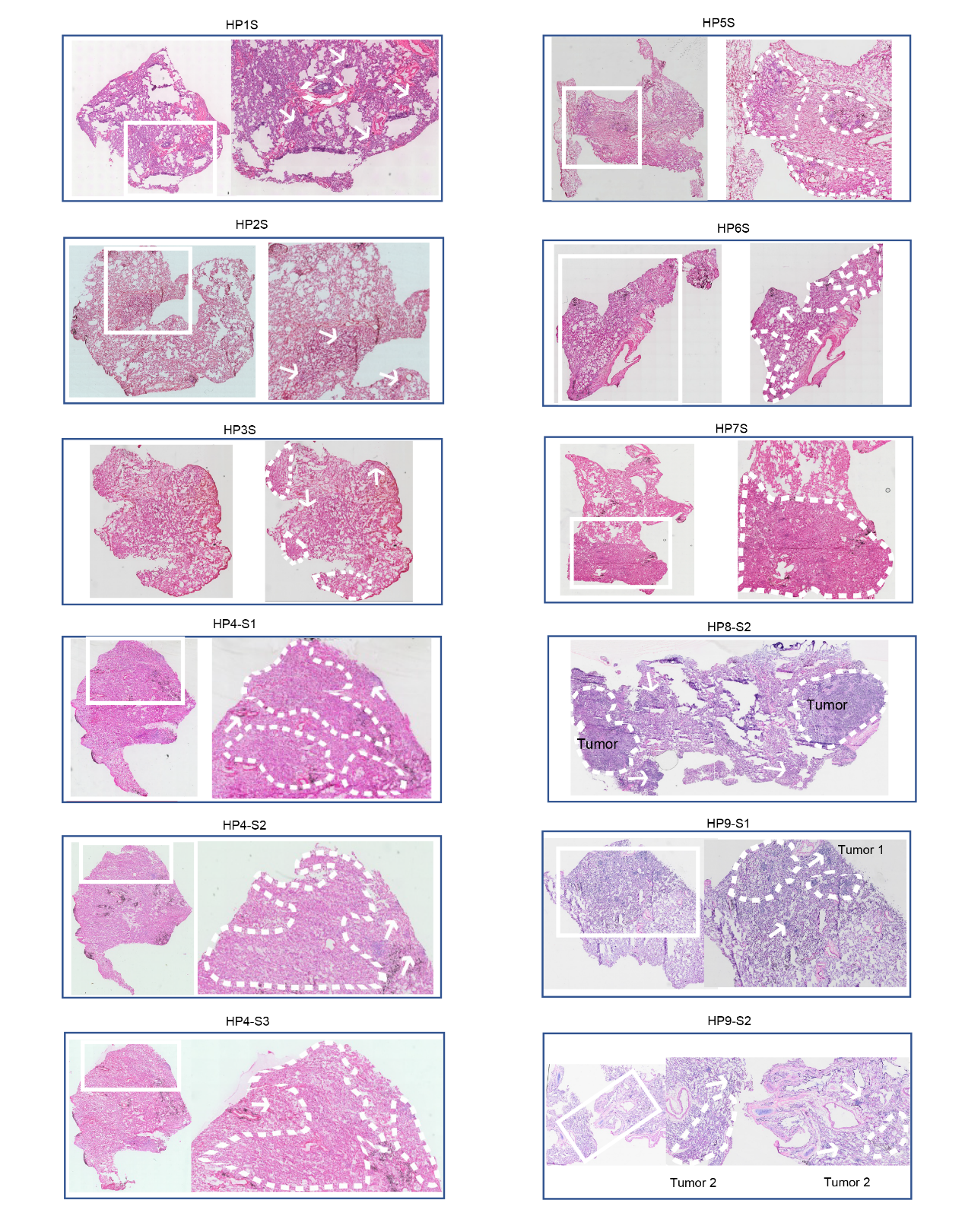


**Supplementary Figure 3. Histopathological annotation of LUAD specimens.** Hematoxylin and eosin (HE) staining was performed on tumor sections, with pathological annotations by two independent pathologists. Boxed regions are shown at higher magnification on the right, with tumor areas delineated by arrows and dashed lines. Patient 4 had multiple sections from a single tumor (HP4-S1, HP4-S2, and HP4-S3). Patient 8 had two distinct tumors (P8T1 and P8T2) analyzed across four sections (HP8-S1, HP8-S2, HP8-S3, and HP8-S4; see Supplementary Figure 1). Patient 9 had three tumors (P9T1, P9T2, and P9T3) mounted on two sections (HP9-S1 and HP9-S2; see Supplementary Figure 1).

**
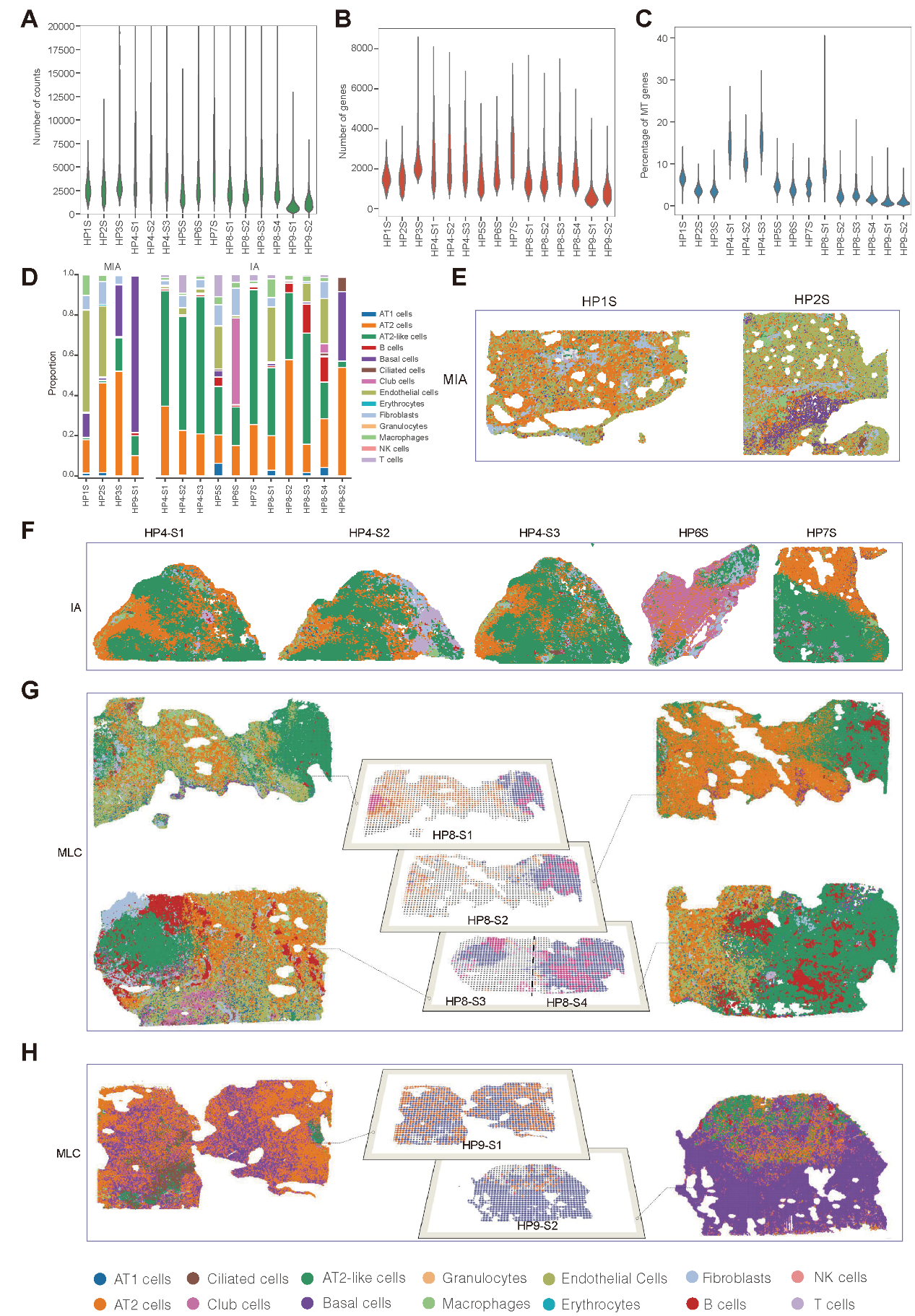
Supplementary Figure 4. Quality control and cellular annotation of spatial transcriptomic data**.

(**A–C**) Violin plots showing quality control metrics across 15 spatially resolved transcriptomic (SRT) datasets from nine LUAD patients: raw unique molecular identifier (UMI) counts per spot (**A**), number of genes detected per spot (**B**), and percentage of mitochondrial (MT) gene expression per spot (**C**). (**D**) Relative proportions of cell populations in MIA (left) and IA (right) tumors across the nine patients. (**E–H**) Spatial distribution of annotated cell populations in representative MIA tumors (**E**), IA tumors (**F**), and multifocal lung adenocarcinoma cases (**G–H**). Corresponding hematoxylin and eosin (HE)-stained sections are shown below each spatial map. DC, dendritic cell; NK, natural killer cell; MLC, multifocal lung cancer.

**
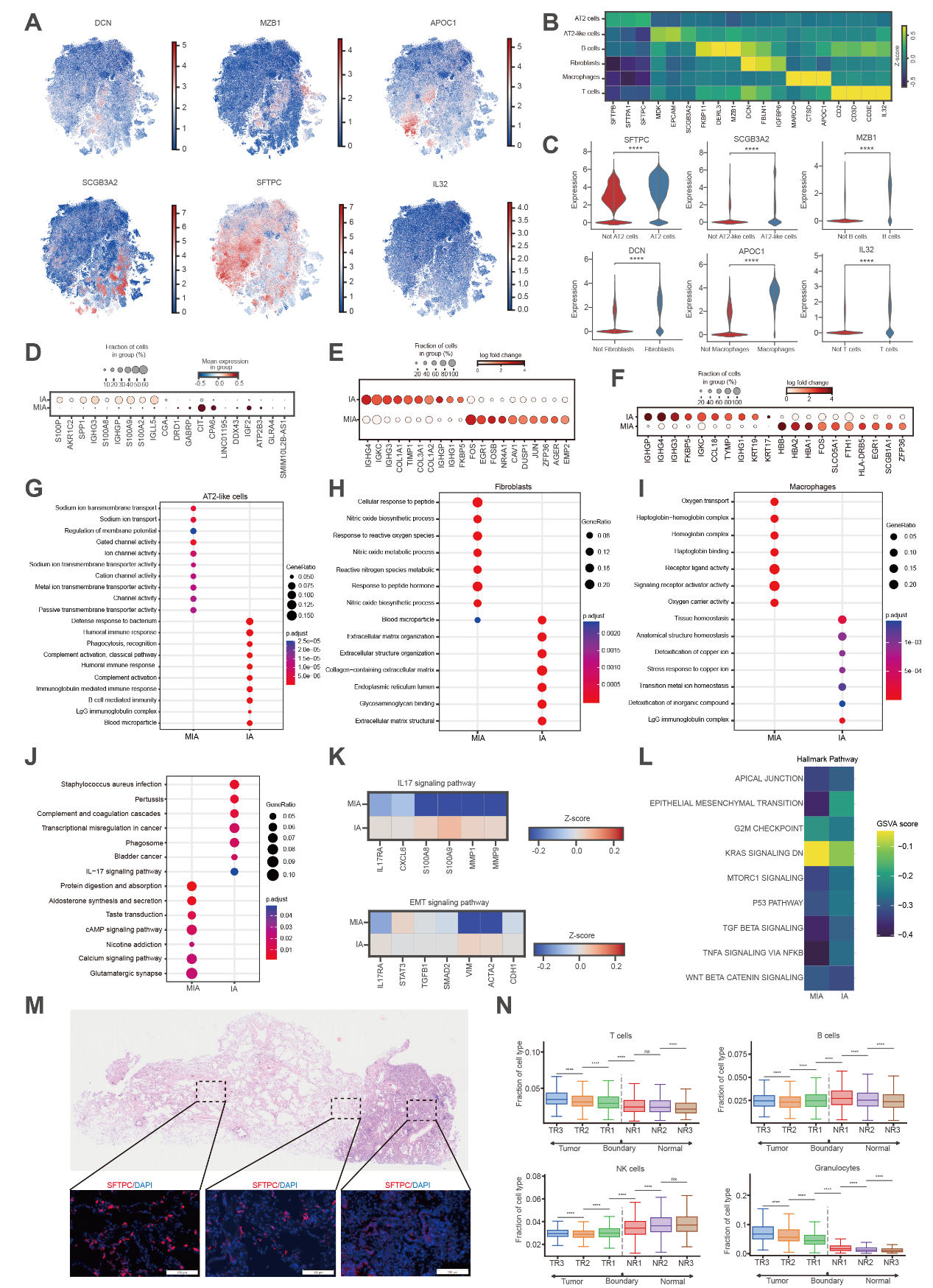
Supplementary Figure 5. Comparative spatial transcriptional analysis of MIA and IA stages.**

**(A)** t-SNE visualization of all Stereo-seq spots colored by expression of six marker genes. **(B)** Heatmap of top differentially expressed genes (DEGs) across spatial clusters from Stereo-seq data. Colors represent z-score scaled expression values. **(C)** Violin plots showing cell type-specific marker gene expression. **(D-F)** Dot plots of the top 10 DEGs in AT2-like cells (**D**), fibroblasts (**E**) and macrophages (**F**) between MIA (n=4) and IA (n=11). **(G-I)** Gene Ontology (GO) enrichment analysis of DEGs in AT2-like cells (**G**), fibroblasts (**H**) and macrophages (**I**) when compared between MIA and IA stages. **(J)** KEGG pathway enrichment analysis of AT2-like cell DEGs between MIA and IA stages. **(K)** Heatmaps showing expression of classic marker genes enriched in IL-17 and EMT signaling pathway in MIA versus IA. **(L)** Gene set enrichment analysis (GSEA) of Hallmark pathways comparing MIA to IA. **(M)** Representative HE and IF staining showing spatial distribution of the AT2 cells (SFTPC red) across multiple regions in patient 7. Nuclei were stained with DAPI (blue). **(N)** Box plots showing average number of T cells, B cells, NK cells and dendritic cells in sequential 500-μm zones extending from the tumor-normal border in patient 7. NR1-NR3: normal regions at 0-500 μm, 500-1000 μm, and 1000-1500 μm from the border, respectively; TR1-TR3: tumor regions at corresponding distances. Cell types were defined as in Figure 2E. Boxplot elements: center line, median; box limits: upper and lower quartiles. Statistical significance assessed by the Wilcoxon rank-sum two-side test (**C, N**). **** p<0.0001. ns, not significant.

**
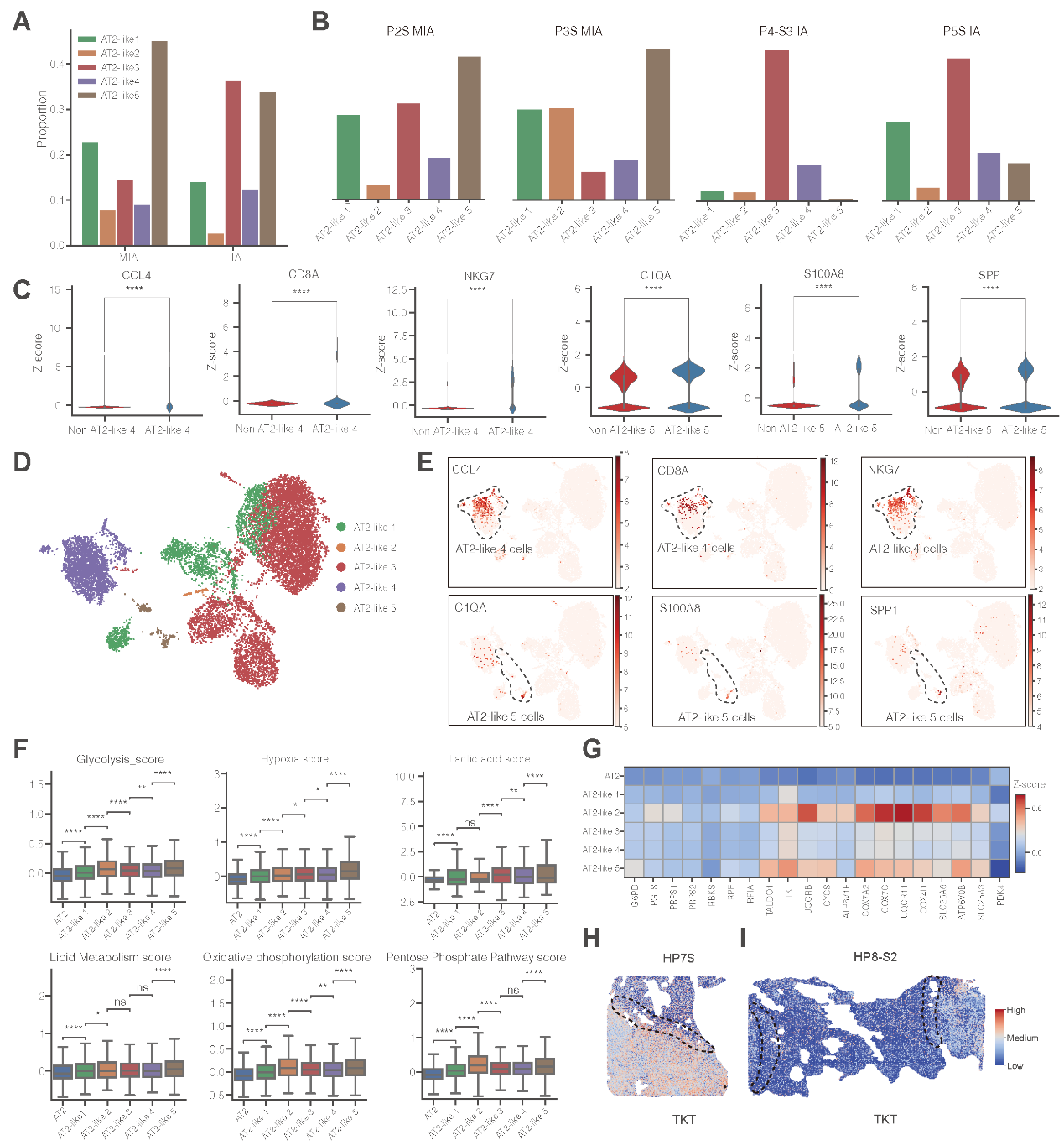
Supplementary Figure 6. Changes in AT2 cells and AT2-like subtypes during progression of LUAD from MIA to IA**.

**(A)** Proportion of AT2-like subtypes in MIA and IA stages across 15 SRT datasets. **(B)** AT2-like subtype composition in patients 2, 3, 4 and 5 from SRT data. **(C)** Expression of immune-related genes in AT2-like 4 and 5 subtypes compared to other AT2-like subtypes from SRT data. **(D)** UMAP visualization of AT2-like subtypes based on single-cell RNA sequencing. **(E)** Heatmap of selected immune-related marker genes across AT2-like subtypes. Colors represent expression levels: red, high; blue, low. **(F)** Boxplots showing pathway activity scores for the indicated biological processes in AT2 cells and AT2-like subtypes. **(G)** Heatmap of pentose phosphate pathway gene expression in AT2 cells and AT2-like subtypes. **(H-I)** The spatial distribution maps colored by TKT expression scores in patient 7 (**H**) and patient 8 (**I**). Boxplot elements: center line, median; box limits, upper and lower quartiles; whiskers, 1.5 × interquartile range (IQR). Statistical significance was assessed by two-sided Mann-Whitney test (**C, F**). * p<0.01, ** p<0.01, **** p<0.0001. ns, not significant.


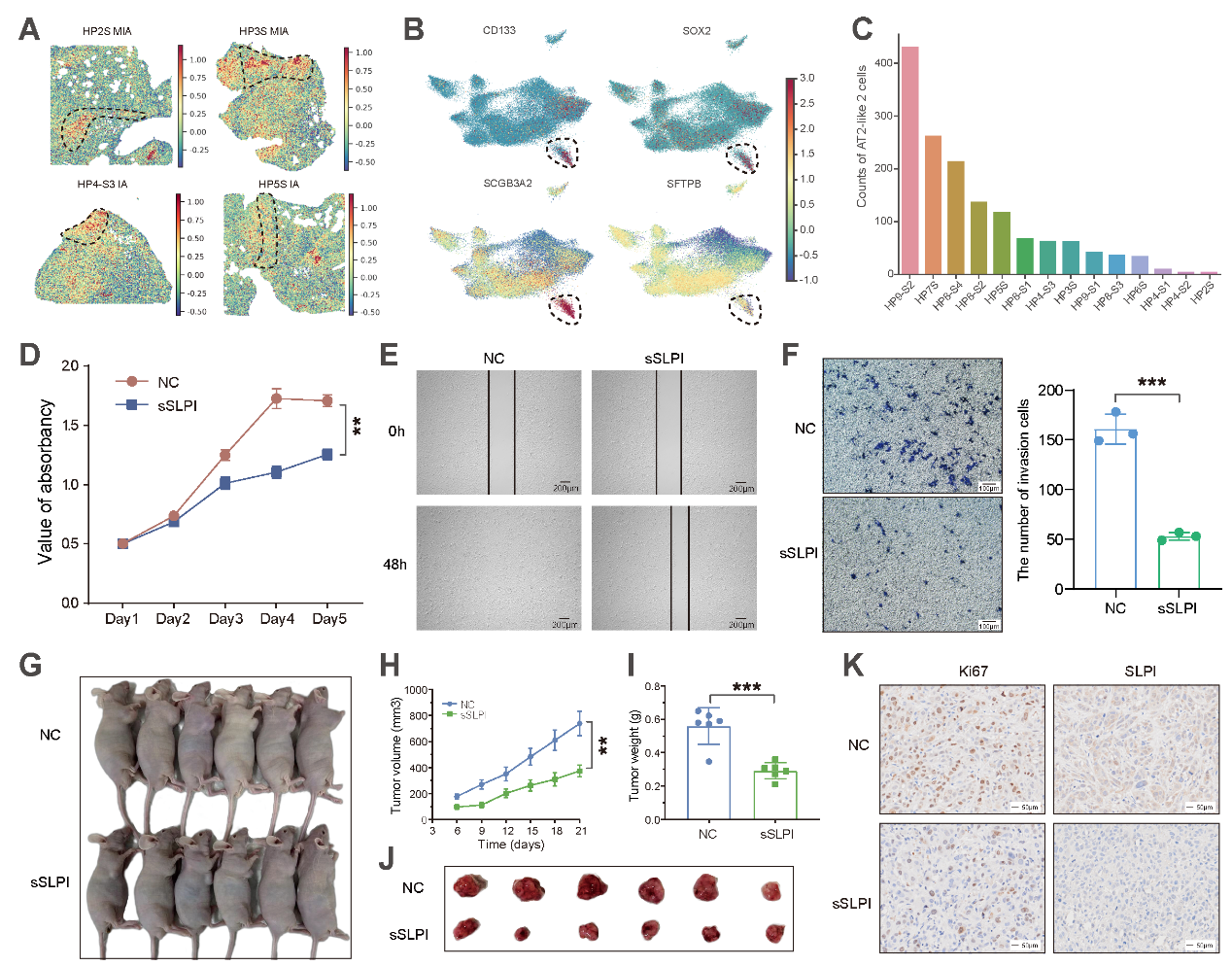


**Supplementary Figure 7. SLPI promotes LUAD cell proliferation, invasion and migration in vitro and in vivo**.

**(A)** Spatial distribution of AT2-like subtypes in IA-stage tumors from patients 2, 3, 4 and 5 based on gene set scores. Dotted lines delineate regions enriched for AT2-like 2 subtypes at the tumor margin. **(B)** UMAP visualization of five AT2-like cell subclusters colored by expression of four stemness-related genes from SRT data. **(C)** Abundance of AT2-like 2 cells across SRT sections from nine patients. **(D)** Cell proliferation kinetics measured by CCK-8 assay in PC9 cells (NC) and SLPI-shRNA PC9 cells (sSLPI) over 5 days (n=3 biological replicates). **(E)** Wound healing assay showing cell migration capability in PC9 cells (NC) and SLPI-shRNA PC9 cells (sSLPI) (n=3 biological replicates). **(F)** Transwell invasion assay comparing invasive capacity of PC9 cells (NC) and SLPI-shRNA PC9 cells (sSLPI) (n=3 biological replicates). **(G)** SLPI knockdown represses tumorigenesis after subcutaneously injecting PC9 cells (NC) and SLPI-shRNA PC9 cells (sSLPI) into nude mice (n=6 mice per group). **(H-I)** Statistical analysis of tumor volume (**H**) and tumor weight (**I**) in nude mice between PC9 cells (NC) and SLPI-shRNA PC9 cells (sSLPI) groups. **(J)** Representative images of xenograft tumors from PC9-NC and PC9-sSLPI groups. **(K)** Immunohistochemical staining of Ki67 and SLPI in xenograft tumor tissues from PC9 cells (NC) and SLPI-shRNA PC9 cells (sSLPI) groups. Representative images shown (n=3 mice per group). Scale bars, 50 μm. Statistical significance was assessed by two-sided Wilcoxon rank-sum test (**D, E**). ** p<0.01, *** p<0.001.


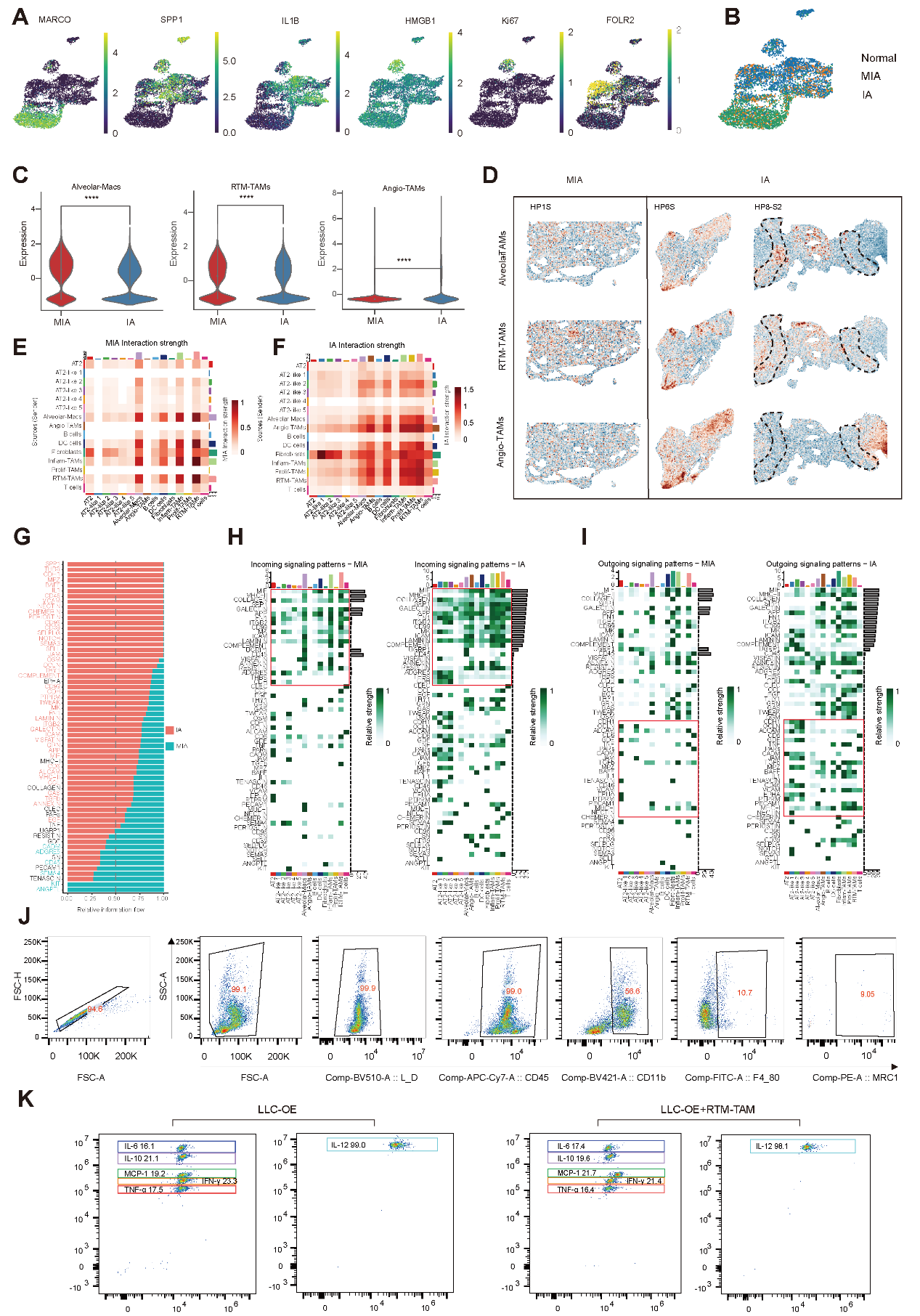


**Supplementary Figure 8. Characterization of macrophages subtypes and intercellular interactions networks in LUAD**.

**(A)** UMAP plot showing the expression of typical markers across five macrophage subtypes. **(B)** UMAP visualization of five macrophage subtypes colored by histological stages (corresponding to Figure 5A). **(C)** Expression levels of subtype-specific marker genes for three macrophage subpopulations in MIA versus IA samples from SRT data. Statistical significance was assessed by two-sided Mann-Whitney-Wilcoxon test. **** p<0.0001. **(D)** Spatial expression patterns of marker genes for Alveolar-Macs, RTM-TAMs and Angio-TAMs in MIA and IA tumors. Dashed lines delineate AT2-like 2 subtype-enriched regions at the tumor margin. **(E-F)** Heatmaps showing the differential cell-cell interaction strength among macrophage subtypes and other cell types in MIA (**E**) and IA (**F**). Top color bars represent aggregate incoming signaling (row sums); right color bars represent aggregate outgoing signaling (column sums). **(G)** Comparison of overall information flow through signaling pathways between MIA and IA tumors. **(H)** Heatmaps comparing incoming signaling patterns between MIA (left) and IA (right) to identify ligand-receptor pairs with divergent activities. **(I)** Heatmaps comparing outgoing signaling patterns between MIA (left) and IA (right) to identify ligand-receptor pairs with divergent activities. **(J)** Flow cytometry gating strategy for sorting RTM-TAMs from mouse lung tissues. Sequential gating: live cells are sorted by L/D, myeloid cells by CD45, macrophages by CD11b and F4/80, and RTM-TAM cells with high MRC-1 expression are obtained. **(K)** Cytometric bead array (CBA) quantification of cytokine levels, including IL-6, IL-10, IL-12, MCP-1, TNF-α and IFN-γ in sorted macrophage populations (n=3 biological replicates per group).

**
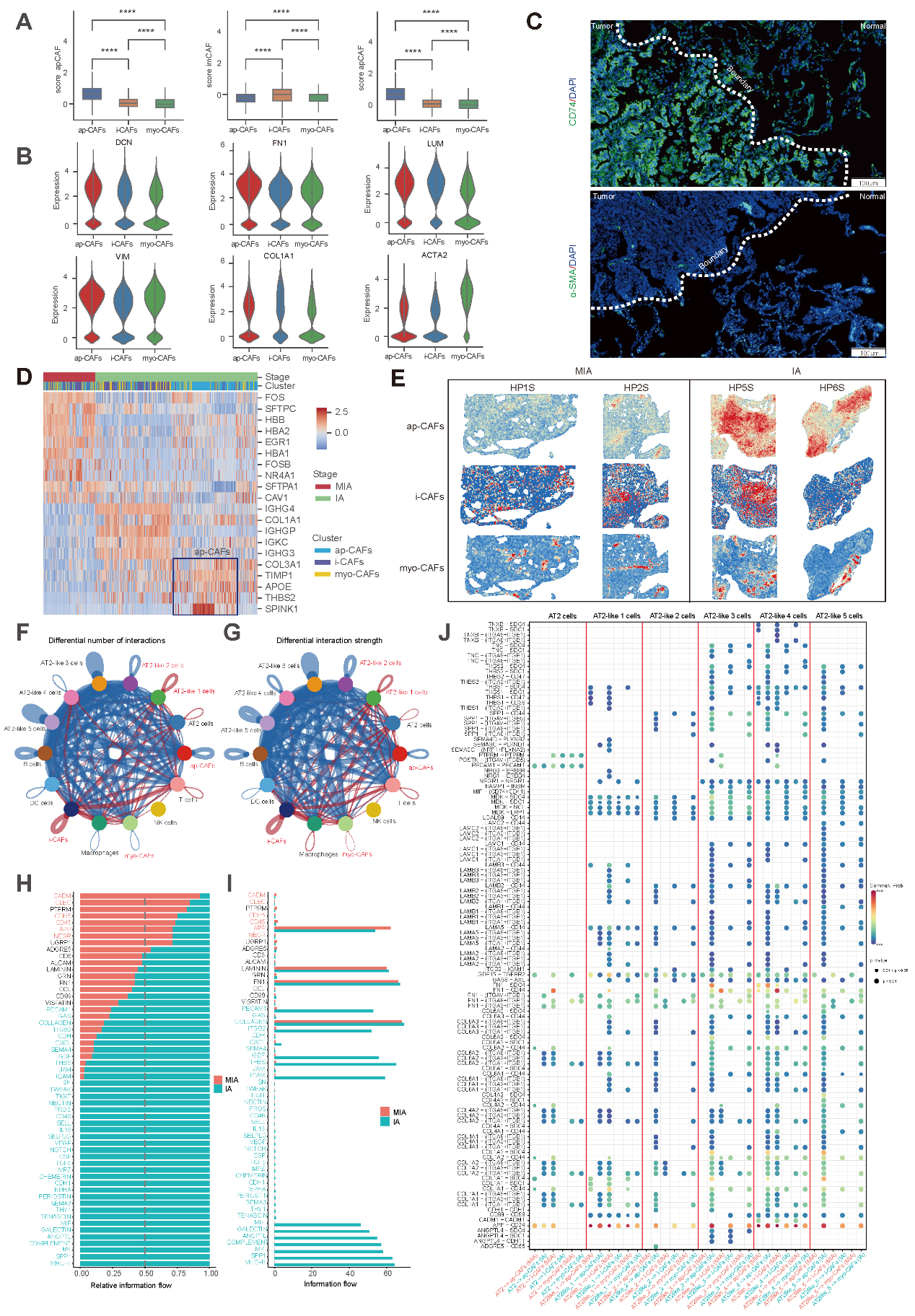
Supplementary Figure 9. Spatial heterogeneity and intercellular communications of fibroblast subtypes in LUAD.**

**(A)** Average expression profiles of differentially expressed gene (DEG) sets across three fibroblast subtypes. Boxplot elements: center line, median; box limits, upper and lower quartiles; whiskers, 1.5 × interquartile range (IQR). Statistical significance was assessed by two-sided Wilcoxon rank-sum test. **** p<0.0001. **(B)** Expression of characteristic marker genes in quiescent fibroblast subsets. **(C)** IF staining showing high expression of ap-CAFs marker CD74 (top) and myo-CAFs marker α-SMA (bottom) in tumor cells within the invasive zone of IA-stage LUAD specimens. Dashed lines delineate the tumor-stroma border. Nuclei were stained with DAPI (blue). Scale bars, 100 μm. **(D)** Heatmap of the top 20 DEGs in fibroblasts between MIA and IA stages from Stereo-seq data. **(E)** Spatial distribution of fibroblast subtypes based on mean expression of different gene signatures. **(F-G)** Circle plots showing differential intercellular communication involving fibroblast subtypes in MIA (**F**) and IA (**G**) tumors. Red (blue) edges represent greater (lower) communication in MIA than in IA tumors. **(H-I)** Comparison of signaling information flow across fibroblasts between MIA and IA stages: Relative disparities in total information flow magnitude (**H**) and aggregate number of active signaling pathways (**I**). **(J)** Communication probability between fibroblast-derived ligands and AT2 or AT2-like cell receptors for selected ligand-receptor pairs.

**Supplementary Table 1.** The clinical and pathological information of 21 LUAD patients

| **Patient ID** | **Single cell barcode** | **Tumor Barcode** | **Sex** | **Age** | **Smoke** | **Tumor Size(cm)** | **Affected lobe** | **Histological Type** | **AJCC Stage** |
| --- | --- | --- | --- | --- | --- | --- | --- | --- | --- |
| Patient1 | / | HP1T | F | 52 | NO | 0.8 | Right middle | MIA | IA1 |
| Patient2 | / | HP2T | M | 52 | NO | 1.1 | Left upper | MIA | IA2 |
| Patient3 | / | HP3T | F | 51 | NO | 1.3 | Right lower | MIA | IA2 |
| Patient4 | / | HP4T | F | 57 | NO | 3.4 | Right upper | IA | IB |
| Patient5 | / | HP5T | F | 44 | NO | 2.1 | Right upper | IA | IA3 |
| Patient6 | / | HP6T | M | 52 | 30y*20 | 4.1 | Left upper | IA | IIA |
| Patient7 | / | HP7T | M | 71 | 40y*3 | 2.5 | Left upper | IA | IB |
| Patient8 | / | HP8T1 | M | 68 | NO | 2.3 | Right upper | IA | IIB |
| Patient8 | / | HP8T2 | M | 68 | NO | 2.3 | Right upper | IA | IA3 |
| Patient9 | / | HP9T1 | F | 53 | NO | 1.5 | Right lower | MIA | IA2 |
| Patient9 | / | HP9T2 | F | 53 | NO | 1.5 | Right upper | IA | IA2 |
| Patient9 | / | HP9T3 | F | 53 | NO | 2.6 | Right upper | IA | IA3 |
| Patient10 | P1T | HP10T | F | 55 | NO | 1.5 | Right middle | IA | IA2 |
| Patient11 | P6T2 | HP11T | F | 45 | NO | 1.5 | Left upper | IA | IA2 |
| Patient12 | P8T1 | HP12T | F | 58 | NO | 1.2 | Left upper | IA | IA2 |
| Patient13 | P12T | HP13T | F | 48 | NO | 4.1 | Left lower | IA | IIIA |
| Patient14 | P13T | HP14T | F | 78 | NO | 3.1 | Right upper | IA | IIA |
| Patient15 | P17T | HP15T | F | 38 | NO | 2.7 | Left lower | IA | IB |
| Patient16 | P18T | HP16T | M | 61 | 20y*20 | 2 | Right upper | IA | IB |
| Patient17 | P20T | HP17T | M | 60 | 30y*20 | 1.4 | Left lower | IA | IA2 |
| Patient18 | P4T | HP18T | M | 60 | 15y*10 | 1.5 | Left upper | MIA | IA1 |
| Patient19 | P5T2 | HP19T | F | 44 | NO | 1.2 | Right lower | MIA | IA1 |
| Patient20 | P6T1 | HP20T | F | 45 | NO | 1 | Left lower | MIA | IA1 |
| Patient21 | P7T2 | HP21T | F | 39 | 15y*2 | 0.7 | Right upper | MIA | IA1 |

**Supplementary Table 2.** The spatial transcriptomic sequencing information of 9 LUAD patients.

| **Patient ID** | **Section Barcode** | **Total reads (GB)** | **Mean Counts (per bin)** | **Median Counts (per bin)** | **Mean Gene Type (per bin)** | **Median Gene Type (per bin)** |
| --- | --- | --- | --- | --- | --- | --- |
| Patient 1 | HP1S | 17.96 | 2,706 | 2,567 | 1,536 | 1,500 |
| Patient 2 | HP2S | 19.34 | 2,739 | 2,596 | 1,485 | 1,472 |
| Patient 3 | HP3S | 25.73 | 4,149 | 3,126 | 2,008 | 1,794 |
| Patient 4 | HP4-S1 | 11.96 | 5,482 | 4,296 | 2,306 | 2,062 |
| Patient 4 | HP4-S2 | 11.97 | 6,194 | 4,697 | 2,466 | 2,184 |
| Patient 4 | HP4-S3 | 11.3 | 5,156 | 4,187 | 2,214 | 2,012 |
| Patient 5 | HP5S | 22.67 | 2,871 | 2,260 | 1,519 | 1,358 |
| Patient 6 | HP6S | 6.31 | 3,929 | 2,987 | 2,430 | 2,164 |
| Patient 7 | HP7S | 6.2 | 7,170 | 5,809 | 3,070 | 2,952 |
| Patient 8 | HP8-S1 | 12.46 | 3,195 | 2,413 | 1,523 | 1,345 |
| Patient 8 | HP8-S2 | 17.19 | 2,779 | 2,183 | 1,528 | 1,368 |
| Patient 8 | HP8-S3 | 11.73 | 6,129 | 3,963 | 2,416 | 2,071 |
| Patient 8 | HP8-S4 | 13.11 | 3,004 | 2,550 | 1,744 | 1,608 |
| Patient 9 | HP9-S1 | 15.67 | 1,293 | 1,156 | 877 | 812 |
| Patient 9 | HP9-S2 | 17.72 | 855 | 696 | 614 | 531 |
